# Tcf-1 protects anti-tumor TCR-engineered CD8^+^ T-cells from GzmB mediated self-destruction

**DOI:** 10.1007/s00262-022-03197-2

**Published:** 2022-04-23

**Authors:** Brendan Zangari, Takemasa Tsuji, Junko Matsuzaki, Hemn Mohammadpour, Cheryl Eppolito, Sebastiano Battaglia, Fumito Ito, Thinle Chodon, Richard Koya, A. J. Robert McGray, Kunle Odunsi

**Affiliations:** 1 Department of Immunology, Roswell Park Comprehensive Cancer Center, Buffalo, NY, USA; 2 University of Chicago Medicine Comprehensive Cancer Center, 5841 S Maryland Ave, Chicago, IL 60637, USA; 3 Department of Obstetrics and Gynecology, University of Chicago, Chicago, IL, USA; 4 Department of Genetics and Genomics and Bioinformatics, Roswell Park Comprehensive Cancer Center, Buffalo, NY, USA; 5 Keck School of Medicine, University of Southern California, Los Angeles, CA, USA

**Keywords:** Cell-therapy, TCF-1, Granzyme B, Cell cycle

## Abstract

**Background:**

T-cell longevity is undermined by antigen-driven differentiation programs that render cells prone to attrition through several mechanisms. CD8 ^+^ T cells that express the Tcf-1 transcription factor have undergone limited differentiation and exhibit stem-cell-like replenishment functions that facilitate persistence. We engineered human CD8 ^+^ T cells to constitutively express Tcf-1 and a TCR specific for the NY-ESO-1 cancer-associated antigen. Co-engineered cells were assessed for their potential for adoptive cellular immunotherapy.

**Methods:**

Tcf-1 mRNA encoding TCF-1B and TCF-1E isoforms, along with GzmB expression were assessed in CD62L ^+^ CD57 ^−^, CD62L ^−^ CD57 ^−^, and CD62L ^−^ CD57 ^+^ CD8 ^+^ T cells derived from normal donor lymphocytes. The impact of stable Tcf-1B expression on CD8 ^+^ T-cell phenotype, anti-tumor activity, and cell-cycle activity was assessed in vitro and in an in vivo tumor xenograft model.

**Results:**

TCF-1B and TCF-1E were dynamically regulated during self-renewal, with progeny of recently activated naïve T cells more enriched for TCF-1B mRNA. Constitutive TCF-1B expression improved the survival of TCR-engineered CD8 ^+^ T cells upon engagement with tumor cells. Tcf-1B prohibited the acquisition of a GzmB ^High^ state, and protected T cells from apoptosis associated with elicitation of effector function, and promoted stem cell-like characteristics.

**Conclusions:**

Tcf-1 protects TCR-engineered CD8 ^+^ T cells from activation induced cell death by restricting GzmB expression. Our study presents constitutive Tcf-1B expression as a potential means to impart therapeutic T cells with attributes of persistence for durable anti-tumor activity.

## Background

In order for adoptively transferred CD8^+^ T cells to mediate durable regression of advanced cancers, they must have sufficient longevity; as not all CD8^+^ T-cell differentiation subsets are capable of long-term persistence as cell-therapy T cells [1–4]. Naïve CD8^+^ T cells exist in a quiescent state in which epigenetic programs imprinted by thymopoiesis restrain activation and potentiate both persistence and readiness for entry into cycling states required for the propagation of large numbers of clonal cells. With activation, naïve CD8^+^ T-cells undergo successive rounds of cell-division to propagate memory T cells with stem-cell-like replenishment functions as well as more differentiated effector cells which are short-lived and function to destroy neoplastic targets via the mobilization of granzymes and IFN-γ. Progressive differentiation through naïve, memory, and effector programs is facilitated by the silencing the locus of T Cell Factor 1 (Tcf-1), a master regulator of T-cell stemness [5, 6]. CD8^+^ T cells in the Tcf-1^+^ stages of differentiation have advanced anti-tumor activity in cellular immunotherapies; Tcf-1-enriched subsets out-persist and out-perform Tcf-1-low/negative subsets in adoptive cell transfer (ACT) [7]. Tcf-1 expression is heterogenous among tumor-infiltrating lymphocytes, and further limited in peripheral blood T cells available for manufacture into CAR or TCR-gene engineered cells [8, 9]. Cell-therapy T cells that do express Tcf-1 are susceptible to *Tcf-1* silencing in vivo, and a consequential loss of cell persistence [10]. A natural question is whether genetic approaches to enforce Tcf-1-mediated programs could prolong the persistence of TCR-engineered CD8^+^ T cells.

TCRs harnessed from naturally occurring tumor-specific T cells allow engineered cell-therapy T cells efficacy against advanced cancers [11], via recognition of HLA-complexed peptide antigenic targets and the transduction of signals that direct cytolytic activity and expansion over attrition. Antigen recognition is understood to impose significant pressure on CD8^+^ T cells. For example, tumor-reactive T cells infiltrating melanoma tumors express Tcf-1 at reduced levels relative to bystander cells lacking tumor specificity [8]. Experiments with TCR-transgenic murine T cells have revealed the potential for differentiation and dysfunction to occur as a result of tumor-recognition rather than just as a result of effects of the tumor microenvironment [12]. With TCR cross-linking, CD3-directed signal transduction cascades elicit activated states which sustain granzyme synthesis [13]. Low intracellular granzyme content is an attribute of stemness and CD8^+^ T cells acquire peak granzyme B (GzmB) expression and cytotoxicity via serial antigen encounter [14]. Granzymes predispose T cells to apoptosis via their leakage from lysosomal granules to the cytoplasm and subsequent cleavage of caspase-3 or disruption of mitochondrial integrity [15–19]. The propensity for granzymes to limit cell longevity is underlined by the finding that GzmB^−/−^ CD8^+^ T-cells have enhanced anti-leukemia activity in pre-clinical models as a result of their being better able to persist to use IFN-γ and FAS-ligand to neutralize cancer cells [17]. Epigenetic regulation of granzyme expression could be a critical determinant of CD8^+^ T-cell longevity. The GzmB locus of Tcf-1-express-ing stem-cell-like CD8^+^ T-cells is inaccessible and further regulated in memory T cells relative to effector cells [20].

In order to mitigate T cell death and enhance anti-tumor activity, T cells transferred into patients may be supported with exogenous IL-2, but off-target activation of self-directed CD8^+^ T-cells and regulatory CD4^+^ T-cells limit the efficacy of IL-2 and there is a recognized need for alternative methods to support cells in vivo [21]. T cell subsets differ in IL-2 receptivity [22], production [23], and sensitivity to deprivation [24]. The potential for a relationship between differentiation subsets and IL-2 requirements compel inquiry into whether Tcf-1 sustains viability in contexts where exogenous IL-2 is not provided. Further compelling is the question of whether Tcf-1 influences cell-cycle activity supported by IL-2.

Herein, we examined the kinetics of Tcf-1 expression in relation to primary activation and found that activation resulted in upregulation of a specific isoform of Tcf-1; TCF-1B. We assessed the impact of constitutive TCF-1B expression on the functions of human CD8^+^ T-cells also engineered to express a therapeutic TCR specific to NY-ESO-1, a particularly immunogenic and widely expressed cancer-associated-antigen. Tcf-1 transgenic T-cells demonstrated resistance to apoptosis associated with the elicitation of cytotoxic functions, modestly enhanced survival in the absence of IL-2 support, and stem-cell-like cycling characteristics of early lineage T-cells. These findings elucidate mechanisms by which Tcf-1-expressing CD8^+^ T-cells persist and implicate Tcf-1 overexpression as a potential strategy for imparting cells with Tcf-1-programmed attributes for adoptive cellular immunotherapy.

## Materials and methods

### Flow cytometry

To detect Tcf-1, surface-marker labelled cells were fixed and permeabilized with True Nuclear Transcription Factor Buffer Set (BioLegend #424,401) and labelled intracellularly with αTcf-1 clone C63D9 (Cell Signaling Technologies #6709). LSR-II and Fortessa cytometers were used. In experiments detecting apoptosis, cells were labelled for fifteen minutes at room temperature with Annexin-V:APC and Zombie UV viability dye in annexin-binding buffer(Biolegend #640,919) (Biolegend #42,307). Data were analyzed with BD Biosciences Flowjo software. Information regarding all antibodies can be found in the Supplemental Methods.

### Quantitative PCR

RNA was extracted using phenol–chloroform extraction and converted to cDNA with Iscript Reverse Transcription Supermix(BioRad #1,708,840). cDNA was assayed by real-time PCR with custom primers from Integrated DNA Technologies and iQSYBR Green Supermix(BioRad #1,708,880), using a BioRadCFX96-Touch-Detection-System: (i) 3-min at 95 °C (ii) 15-s at 94 °C, detection (iii) annealing and extension for 1-min (iv) repeat ii-iii 39 times. Data were analyzed with BioRad CFX Manager. Expression was evaluated using Ribosomal Protein L4 (RPL4) as a control gene. Relative expression values (y) used for comparison were generated by subtracting the Cq of RPL4 from the Experimental Cq and log-transforming the difference with *y* = 2^−ΔCq^. Primer information and annealing temperatures are found in Supplemental Methods.

### Cloning TCF-1-GFP transgenes

cDNA from Jurkat cells was used as a template for PCR to generate amplicons for assembly. Primers were designed for TCF-1B (**NM_003202.5**) (**NP_003193.2**). dnTCF-1B (**NM_201632.5**)(**NP_963963.1**) was amplified from exons 2–10 of TCF-1B. TCF-1E (**EAW62279.1**) was generated by assembly of exons 2–8 with a gBlock^Tm^ gene fragment for exons 9–10. Detailed methods are in Supplemental Methods.

### Tcf-1 and TCR transduction

A high-affinity NY-ESO-1 specific HLA-A*02-restricted TCR gene was transduced, 19305DP (19,305-TCR) [25]. CD8^+^ T cells were isolated from cryopreserved healthy-donor blood mononuclear cells (PBMC) using a Naive CD8 + T-cell Isolation-Kit (Miltenyi Biotec 130–093-244). The isolated cells (2.0 × 10^6^ cells/well) < space removed were plated in complete RPMI supplemented with recombinant human IL-2* 300 IU/ml (Peprotech 200–02) and anti-CD3/CD28 dynabeads (1:3 to 1:1, bead:cell) (Thermo Fisher Scientific 11161D) in 6-well culture plates. After 48 h, T cells were harvested and seeded onto plates coated with recombinant retrovirus from PG13 supernatant. After transduction, T cells were de-adhered from dynabeads and re-suspended at (0.5–0.7 × 10^6^/ml) in complete RPMI with IL-2. *Media supplemented with IL-2, IL-7, and IL-15 (10 ng/ml, Peprotech 200–02, 200–07, 200–15) was used for transduction of PBMC and naive CD8^+^ T-cells.

### PBMC GzmB content and Cytokine production

PBMC were transduced and expanded 9-days and stained to detect GzmB (BD Bioscience 560,213). PBMC were expanded an additional 9-days with media supplemented with solubilized anti-CD3 antibodies (OKT3) (50 ng/ml) and IL-2 (300 IU/ml). PBMC were re-stimulated with Cell Activation Cocktail with BFA for detection of IL-2 and IFN-γ (Biolegend).

### In vivo Anti-tumor Efficacy

NSG mice (NOD.Cg-Prkdc^Scid^ IL2rg^Tm1wjl^/SzJ Jackson Laboratory #005,557) were used. HLA-A*02:01^+^ NY-ESO-1^+^ SK-MEL-37 melanoma cells (1 × 10^6^) were injected subcutaneously into the left rear flank. Mice with tumors that reached 40 mm^3^ were injected intravenously with human CD8^+^ T cells (0.75 × 10^6^) transduced to express either [19305-TCR and GFP] or [19305-TCR and TCF-1B-GFP]. IL-2 was delivered via intraperitoneal injection on the day of transfer and 24- and 48-h after (50,000 IU GoldBio IL-2). Tumors were measured with calipers, measuring the longest dimension (L) and perpendicular width (W):Volume (mm^3^) = (L × W^2^)/2. Tumor-infiltrating lymphocytes were extracted from tumors via enzymatic digestion with type IV-collagenase(Sigma#c5138-500 mg) and typeIV-DNAse1(Sigma#D5025-15ku). Methods for analysis of tumor-infiltrate are in Supplemental Methods.

### Cytotoxicity assays

TCR-engineered CD8^+^ T-cells (4 × 10^5^ cells) were plated on SK37 (5 × 10^4^ cells) in the 48-well format. After 24-h cells were harvested for flow-cytometry. Adherent cells were further harvested using 0.25% trypsin/EDTA solution. In the second format, used to study GzmB upregulation, T cells (2 × 10^5^) were seeded onto SK37 (1.5 × 10^4^) in 96 well plates. Cultures were collected after 15-h. Some cultures were supplemented with BFA at 3 μg/ml (ThermoFisherScientific#00–4506-51).

### Survival and expansion co-cultures

19,305-TCR engineered CD8^+^ T-cells (2.0 × 10^5^ cells) expressing GFP or Tcf-1 were suspended in 200 μL media and passaged onto SK37 (1.5 × 10^4^) pre-seeded in the 48-well format. The media in which T cells were suspended contained either no IL-2 (survival cultures) or IL-2 at concentrations of 300 or 1500 IU/ml (expansion cultures). After 72-h, media was replenished. After 7-days, cells were collected for cytometry. Countbrite Absolute Counting beads were used to determine relative numbers of CD45^+^ T cells(ThermofisherScientific#C36950).

### Statistical analyses

Graphpad Prism.v9 was used to assess statistical significance. Student *t*-tests were used to compare significance, with *p*-values of less than 0.05 represented by an asterisk. All graphs include error-bars which indicate one standard deviation about the mean.

## Results

### Dynamic regulation of Tcf-1 and GzmB in relation to CD8^+^ T-cell differentiation

**Fig. 1:**
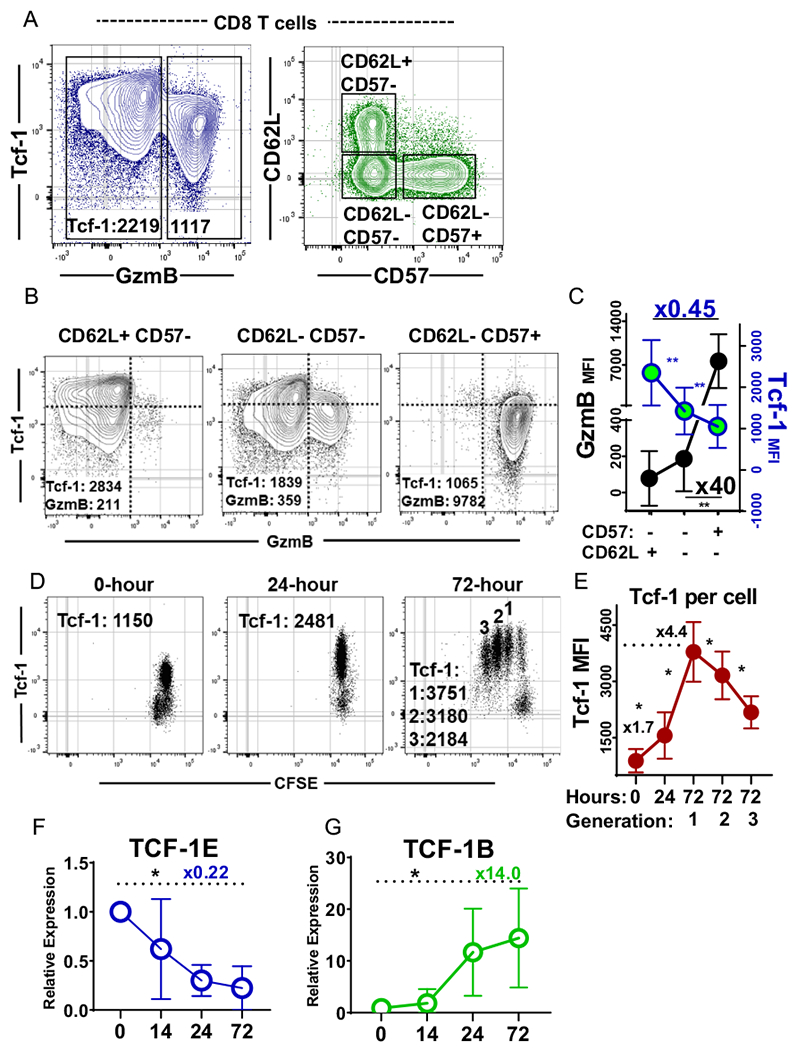
Tcf-1 and GzmB index CD8^+^ T cell differentiation **A** CD8^+^ T cell differentiation subset expression of Tcf-1 & GzmB. Cells from the blood of healthy donors were assayed. In the left plot, gates were applied based on observed differences in GzmB content. Tcf-1 median fluorescence intensity is listed for each population within the gate. In the plot to the right, subsets were defined as (i) CD62L+ CD57− (ii) CD62L− CD57− (iii) CD62L− CD57+ . The gates used to assay CD8^+^ T cells are seen in Supplemental Fig. 1A. Gates are superimposed on the plots for the purpose of enhancing resolution. **B** Differentiation subset expression of Tcf-1 & GzmB, from subsets defined in (**A**). Median fluorescence intensity is noted for the entirety of cells in the plot. A blue axis is overlaid for visual enhancement. **C** Expression of Tcf-1 & GzmB, by CD8^+^ T cells at different stages of differentiation, as defined in (**A**). Tcf-1 is plotted in blue & green and GzmB is plotted in black. The left axis in black is for GzmB and the right axis in blue is for Tcf-1. Comparison of CD62L^−^CD57^+^ T celfs to CD62L^+^ CD57^−^ T cells revealed the later subset to express Tcf-1 at 0.45-fold lower levels than the earlier, as noted. Comparison of CD62L^−^CD57^−^ to CD62L^−^CD57^+^ cells revealed the latter to express GzmB at 40-fold greater levels than the earlier. Paired *t*-tests were applied to assess statistical significance. *N* = 6 donors. Asterik(s) indicate comparisons which yielded a *p*-value of less than 0.05. **D** Tcf-1 content of initial progeny of naïve human CD8^+^ T cells. Naïve T cells were purified from cryopreserved PBMC, labelled with CFSE, and activated with anti-CD3/CD28 dynabeads and IL-2 (300 IU/ml). Cells were assayed for Tcf-1 content and division at various timepoints. Median fluorescence intensity is noted on each plot. In the right-most plot, for cells assayed after 72 h, three populations were examined: cells that had undergone one, two, or three divisions. Gates are omitted, for the purpose of enhancing visualization of each population. Gates used may be viewed in Supplemental Fig. 1. **E** Tcf-1 content in relation to primary activation; dynamic expression of Tcf-1 in relation to naive CD8^+^ T cell activation, quantified from (**D**). Tcf-1 is quantified for (i) naïve T cells assayed immediately, “0” (ii) cells activated for 24 h, “24“ (iii) the progeny of naïve T cells resulting from 1, 2, or 3 rounds of cell division over 72 h: “72:1” “72:2” “72:3”. Median fluorescence intensity (MFI) was compared using paired *t*-tests. An asterisk indicates a comparison which yielded a *p*-value of less than 0.05. **F** Expression of E-isoform mRNA transcripts in relation to primary activation, using RPL4 as a house-keeping gene. E-isoform coding sequences are produced from the splicing of exon-8 to exon-9 and splicing of exon-9 to the three-prime splice acceptor found in exon 10. They were assayed using a qPCR assay characterized in Supplemental Fig. 1B, for *N*=9 donors. A paired T*T*test was used to assess statistical significance with asterisk(s) indicative of a comparison which yielded a *p*-value of less than 0.05. **G** Expression of B-isoform mRNA transcripts in relation to primary activation, using RPL4 as a house-keeping gene. B-isoform coding sequences are produced from the splicing of exon-8 to the exon-10 splice acceptor (3’). They were assayed using the qPCR assay characterized in Supplemental Fig. 1B, for *N* = 9 donors. A paired *T*-test was used to assess statistical significance, with asterisk(s) indicative of a comparison which yielded a *p*-value of less than 0.05

CD8^+^ T-cell differentiation is tightly regulated to ensure some cells persist to fulfill progenitor roles while others function to engage in immuno-surveillance and cytotoxic activity [26]. While antigen-experience is understood to be pre-requisite for expression of GzmB, it remains unclear whether regulation of GzmB also occurs in the stages of differentiation subsequent to primary activation, in which memory T-cells with progenitor functions are maintained by Tcf-1 mediated programs [26]. Therefore, to begin our study, we examined the degree to which subsets of CD8^+^ T-cells in different stages of differentiation co-express Tcf-1 and GzmB. CD8^+^ T-cells of normal donor blood formed discrete populations that expressed Tcf-1 and GzmB at different levels (Fig. 1A, left plot). We stratified three subsets for comparison of Tcf-1 and GzmB enrichment: (i) CD62L^+^CD57^−^ T-cells which include naïve and central memory T-cells expected to express Tcf-1 at peak levels (ii) CD62L^−^CD57^−^ T-cells which include effector and effector-memory T cells (iii) CD62L^−^CD57^+^ T-cells which include senescent effector and effector-memory T cells (Fig. 1A, right plot). These subsets differed with respect to their enrichment for populations expressing Tcf-1 versus GzmB at peak levels (Fig. 1B): CD62L^+^CD57^−^ cells were enriched for a population with peak expression of Tcf-1 and little GzmB. Senescent CD62L^−^CD57^+^ cells formed a discrete population with minimal Tcf-1 content and peak GzmB content. CD62L^−^CD57^−^ cells were heterogenous, containing populations with peak Tcf-1 expression and peak GzmB content. Since GzmB was found at significantly reduced levels in CD62L^−^CD57^−^ cells relative to CD62L^−^CD57^+^ cells (Fig. 1C), sub-maximal GzmB content may be an attribute of antigen-experienced T-cell subsets that express Tcf-1.

Tcf-1 is expressed as one of a number of isoforms which vary as a result of dual promoter usage and alternative splicing [27]. TCF-1B and dnTCF-1B have near-identical sequences except the latter lacks an amino-terminal domain thought to bind *β*-catenin. Compared to TCF-1B, TCF-1E recognizes a wider range of gene-targets via an extended C-terminus [28]. It remains unclear whether these isoforms are of variable importance to naïve versus memory stages of differentiation, and whether they differ in utility to our aims. Therefore, we characterized their expression in relation to primary activation, during which naïve T-cells propagate progeny with peakper-cell Tcf-1 protein levels in the initial rounds of cell division [5]. We isolated naïve human CD8^+^ T-cells and used flow-cytometry to assay Tcf-1 content before activation, after 24-h of activation, and after 72-h of activation. T cells assayed after 24-h had twice the Tcf-1 content of those assayed at baseline and were composed of a single population which had not divided (Fig. 1D). After 72-h, four generations of cells with homogenous Tcf-1 content could be observed. Tcf-1 was found at peak levels in cells that had undergone one division and was found at reduced levels in cells that had divided two to three times, but remained homogenous among cells of a given generation (Fig. 1E). The pattern of expression observed is consistent with a model where naïve cell activation results in upregulation of Tcf-1 protein levels in advance of cell-division, suggestively so sufficient Tcf-1 content exists for assortment into memory T-cells generated by successive rounds of division. This mechanism would effectively prevent cell-division from out-pacing production of Tcf-1 required for daughter cells. We used qPCR to assess changes in isoform abundance in relation to primary activation: TCF-1E-transcripts peaked in naïve cells and were found at significantly reduced levels after activation (Fig. 1F). TCF-1B-transcripts were minimally expressed in naive cells and reached peak levels in cells activated for 72-h (Fig. 1G). These results suggest that TCF-1E primarily serves naïve cell gene-expression programs and that TCF-1B is upregulated to accommodate Tcf-1 expression in memory T cells.

### Tcf-1 overexpression antagonizes human CD8^+^ T-cell GzmB expression

**Fig. 2:**
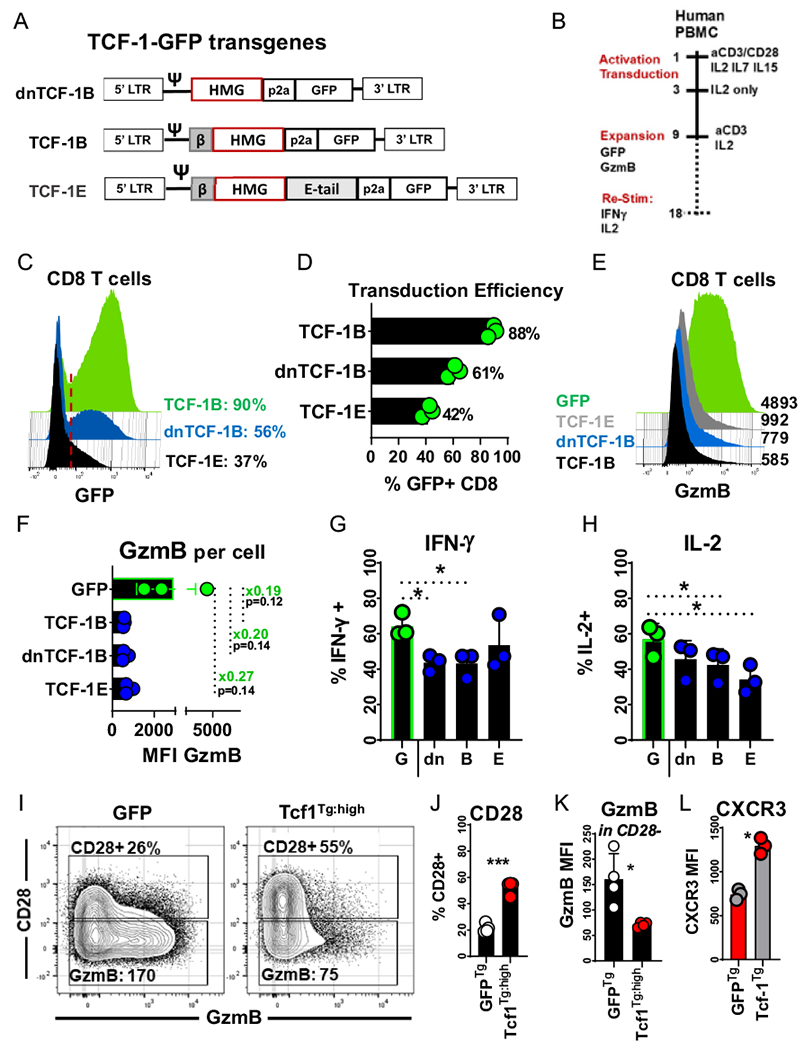
Effect of Tcf-1 on CD8^+^ T cell function and phenotype. **A** Design of recombinant human Tcf-1 transgenes for expression of Tcf-1 and GFP. Each transgene encodes a different isoform of Tcf-1, linked to green fluorescent protein (GFP) by a 2a self-cleaving peptide. Transgenes were designed for expression of three isoforms: dominant negative TCF-1B “dnTCF-1B” which lacks a *N*-terminal transactivation domain, TCF-1B which is canonical, and TCF-1E which contains a C-terminal which differs from TCF-1B and dnTCF-1B. Domains include a N-terminal transactivation domain (*β*), High mobility group DNA binding domain (HMG), extended C-terminal “E-Tail” unique to TCF-1E, self-cleaving peptide, and GFP. **B** Scheme to assess the functionality of CD8^+^ T cells after transduction with one of three TCF-1-GFP transgenes or a GFP control gene, in the context of protocols established for manufacture of gene-engineered T cells for cell-therapy. PBMC were activated with anti-CD3/CD28 dynabeads and briefly cultured in media supplemented with IL-2, IL-7, and IL-15 during transduction. After 3-days, activation beads were separated from T cells and T cells were re-suspended in IL-2 supplemented media for expansion through day nine. After 9-days, GFP and GzmB expression were assessed. Cells were then expanded in media supplemented with solubilized anti-CD3 antibodies (clone OKT3) and IL-2 per methods established in Kochenderfer’s 2009 *Journal of Immunotherapy.* After 18 total days of expansion, cells were re-activated with mitogens for 5-h for assessment of interferon gamma and interleukin-2 production via flow-cytometry. Biolegend Cell-Activation cocktail with brefeldin A was used to re-stimulate T cells. **C** GFP content of human CD8^+^ T cells transduced to express individual Tcf-1-GFP transgenes, as assessed via flow-cytometry. The red line is representative of the threshold used to define GFP positive cells. The efficiency of transduction is noted to the right, as a percentage of CD8^+^ T cells within the PBMC assayed. **D** Transduction efficiency of Tcf-1 transgenes, as assessed by GFP positivity in (C), for transgenic cells from 3 different donors. **E** The impact of Tcf-1 on GzmB content; the GzmB content of Tcf-1 transgenic CD8^+^ T cells was assayed 9-days after the initiation of cell-manufacture. GFP positive CD8^+^ T cells are compared, with median fluorescence intensity noted to the right. T cells were assayed 9 days after the initiation of the scheme in (B). **F** The impact of Tcf-1 on GzmB content; the GzmB content of GFP + CD8^+^ T cells transduced to express different isoforms is compared with a bar graph. A paired *t*-test was used to assess significance. *N* = 3 donors. **G** Impact of Tcf-1 on interferon-gamma production. This bar graph compares frequencies of IFNγ producing cells among GFP + CD8^+^ T cells, among PBMC re-stimulated with Biolegend Cell Activation Cocktail with BFA. “G” indicates GFP control cells and “dn, B, and E” refer to T cells transduced to express GFP transgenes for dnTCF-1B, TCF-1B, and TCF-1E. Cells were assayed after 9 days of culture with anti-CD3 antibody (50 ng/ml OKT3) and IL-2 (300 IU/ml) after 9 days of expansion after activation and transduction. A paired *t*-test was used to assess significance. *N* = 3 donors. Flow-cytometry plots can be seen in Supplementary Fig. 1C. An asterisk indicates a comparison which yielded a *p*-value of less than 0.05. **H** Impact of Tcf-1 on interleukin-2 production, after re-stimulation with Biolegend Cell Activation Cocktail with BFA. GFP + CD8^+^ T Cells were assayed after 9 days of culture with anti-CD3 antibody (50 ng/ml OKT3) and IL-2 (300 IU/ml), after 9 days of expansion after activation and transduction. Flow cytometry plots are found in Supplementary Fig. 1D. A paired *T*-test was used to assess significance, with an asterisk representative of a comparison which yielded a *p*-value less than 0.05 *N* = 3 donors. **I** The effect of the TCF-1B-GFP transgene on phenotype, with respect to expression of CD28 & GzmB after extensive proliferation, with naive CD8^+^ T cells used as starting material. Naïve CD8^+^ T cells (i) were purified from PBMC (ii) activated and transduced to express a Tcf-1 or a GFP control gene (iii) expanded in IL-2 supplemented media. After 7-days, these cells were labelled with Cell-Trace division dye and passaged into cultures with media supplemented with solubilized anti-CD3 antibodies and IL-2 to promote cell division. Five days later, cells were assayed via flow-cytometry to detect CD28 and GzmB. GFP + CD8^+^ T cells with peak Tcf-1 content (4.7-fold greater than GFP controls) deemed “Tcf1^Tg: high^” CD8^+^ T cells were compared to GFP + control CD8^+^ T cells that had identical division history, via the gating strategy seen in Supplemental Fig. 1E. The frequency of CD28 + cells is listed on each plot, as is the median fluorescence intensity of GzmB in CD28− cells. **J** Effect of Tcf-1 on CD28: This is a bar graph that compares the CD28 expression of GFP control cells to that of Tcf1^Tg:High^ CD8^+^ T cells with identical division history, as seen in (I). This graph compares populations via a paired *t*-test, and asterisks indicate comparisons which yielded a *p*-value less than 0.05, for *N* = 4 donors. **K** GzmB content of CD28-populations: This is a bar graph that compares CD28 expression in GFP control cells and Tcf1^Tg:High^ CD8^+^ T cells with identical division history, as seen in (I), with original gates seen in Supplemental Fig. 1E. This graph compares populations with a paired *t*-test. *N* = 4 donors. An asterisk indicates a comparison which yielded a *p*-value below 0.05. **L** CXCR3 expression of Tcf-1 transgenic CD8^+^ T cells at the completion of manufacture, from PBMC. CXCR3 was assayed in GFP + CD8^+^ T cells at the completion of a cell-manufacture protocol akin to those used to prepare gene-engineered T cells for cell-therapy. PBMC were (i) activated with anti-CD3/CD28 dynabeads in media supplemented with IL-2 (ii) transduced to express GFP or the TCF-1B-GFP transgene (iii) expanded for a total of nine days, the shortest amount of time T cells are typically expanded for cell-therapy. *N* = 3 donors. A paired *t*-test was used to assess statistical significance. An asterisk indicates a comparison which yielded a *p*-value below 0.05

We cloned GFP transgenes for constitutive expression of TCF-1B, TCF-1E, and dnTCF-1B (Fig. 2A). We reasoned that one isoform could be advantaged over others and that the activity of each could be assessed via detection of GzmB, IFN-γ, and IL-2 after expanding cells via standard protocols. We assayed CD8^+^ T cell GzmB content after expanding transduced PBMC for a total of nine days, and assayed cytokine-induction with mitogens after an additional nine days of expansion with solubilized anti-CD3 antibodies and IL-2 (Fig. 2B). The transduction efficiency of each isoform was variable, with TCF-1B, dnTCF-1B, and TCF-1E expressed by 90%, 56%, and 37% of CD8^+^ T cells (Fig. 2C–D). Each isoform repressed GzmB, reducing levels to as little as 1/5^th^ of that of GFP transduced control cells on a per cell basis (Fig. 2E), resulting in large but non-significant differences (Fig. 2F). Surprisingly, each isoform was observed to antagonize cytokine production (Fig. 2G, H). Since TCF-1B was the isoform relevant to antigen-experienced stages of differentiation, it was selected for further characterization, and cells transduced with it will hereafter be referred to as Tcf-1 transgenic (Tcf1^Tg^).

CD28 is not only expressed by naive T cells but also effector and effector-memory T cells which have not undergone terminal differentiation [29]. Since CD28 is increasingly being used to define T cells which have yet to reach senescence, we examined the ability of Tcf-1 to maintain CD28^+^ cells [30, 31]. Naïve CD8^+^ T cells were (i) transduced and expanded nine days in IL-2 supplemented media (ii) labelled with proliferation dye (iii) further expanded via culture with media supplemented with solubilized anti-CD3 antibodies and IL-2. We compared the CD28 and GzmB content of GFP^+^ Tcf1^Tg^ T-cells with peak Tcf-1 expression to GFP^+^ control T cells (GFP^Tg^) with similar division history. Tcf-1 maintained a CD28^+^ population that had low GzmB content relative to CD28^−^ cells (Fig. 2I, J). CD28^−^ GFP^Tg^ T-cells had three-fold greater GzmB levels than CD28^−^ Tcf1^Tg^ T-cells (Fig. 2K), indicating that Tcf-1 could limit the cytotoxic potential of CD8^+^ T cells. CXCR3 promotes efficient trafficking to tumors and its expression could be a desirable attribute for cell-therapy T cells [32]. Further, CXCR3 is expressed in a hierarchical manner by early lineage differentiation subsets [33]. Therefore, we assayed CXCR3 in Tcf1^Tg^ CD8^+^ T-cells after nine days of cell manufacture from PBMC, the earliest time point at which gene-edited cells are typically injected after in vitro expansion. CXCR3 was significantly enriched in Tcf1^Tg^ CD8^+^ T-cells relative to control cells (Fig. 2L).

### Tcf-1 allows TCR-engineered T cells to resist death associated with elicitation of cytotoxicity

**Fig. 3:**
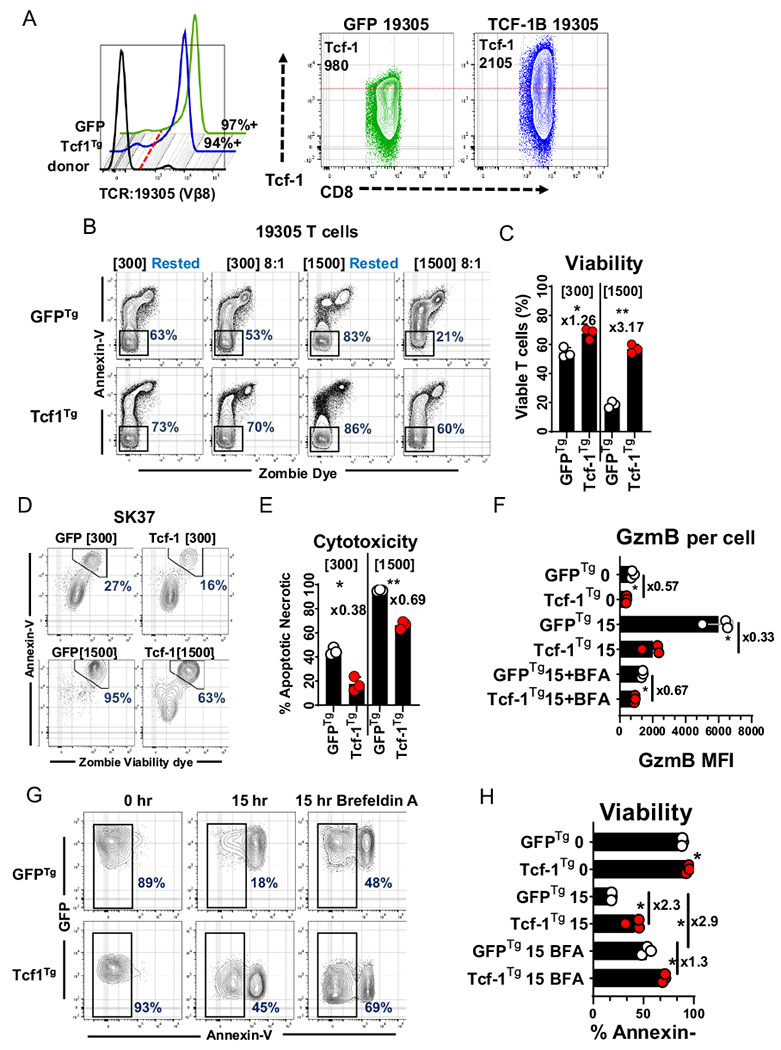
Tcf1^Tg^ TCR-engineered CD8^+^ T cells resist activation induced cell death **A** Expression of the anti-NY-ESO-1 TCR and Tcf-1 in engineered CD8^+^ T cells. To the left, a flow-cytometry histogram depicts labelling of the 19,305-TCR with antibodies specific to Vβ8. T cells transduced to express the TCR and GFP (green histogram) are compared to T cells transduced to express the TCR and TCF-1B-GFP (blue histogram) as well as cells from the same donor which had not been transduced (black histogram). The red dotted line is representative of the threshold used to assign positivity. To the right is a set of plots depicting the Tcf-1 content of the cells, without gating on GFP or Vβ8. A red dotted line is overlaid to allow visualization of differences in Tcf-1 expression. The median fluorescence intensity of Tcf-1 [for the entirety of the cells on the plots] is in the top-left corner. **B** T cell survival of a 24-h engagement with SK37, after conditioning with IL-2 and serial antigen exposure. T cells were labelled with annexin-v and viability dye after 24-h of co-culture with SK37. Gates capture cells which have not labelled with dye or annexin-v and which therefore have optimal viability. The CD8^+^ T cells used for this experiment were derived from those characterized in (A): cells in (A) were conditioned via propagation over SK37 monolayers, with passage once every 7-days for 21-days, in media supplemented with either 300 or 1500 IU/ml IL-2. After 21-days, cells were implemented in this assay. Rested T cells were seeded into wells not containing SK37. Representative gates were superimposed, for optimal resolution. T cells were stratified from SK37 via both forward-scatter and side-scatter profile as well as expression of CD45, as seen in Supplemental Fig. 2A. **C** T cell survival of a 24-h engagement with SK37. The frequency of viable T cells from the gate applied in (B) is quantified in this graph. *N* = 3 technical replicates. *T*-tests were applied without pairing. Asterisk indicate comparisons which yield a *p*-value of less than 0.05. **D** Cytotoxicity of TCR-engineered cells: SK37 labelling with annexin-v and zombie viability dye after 24-h of co-culture with T cells, the assay from (B). Gates capture cells with peak labelling for annexin-v and zombie UV dye, which are apoptotic and or necrotic. **E** Cytotoxicity of TCR-engineered cells: Viability of SK37 after co-culture with T cells for 15-h. *N* = 3 technical replicates. *t*-tests were applied without pairing. Asterisks indicate comparisons which yielded a *p*-value of less than 0.05. **F** GzmB synthesis by TCR-engineered T cells in response to SK37. TCR-engineered CD8^+^ T cells were cultured with SK37 and assayed for GzmB expression under several conditions: (i) immediately after the initiation of cultures “0” (ii) after 15 h of co-culture “15” (iii) or after 15 h of co-culture in media supplemented with BFA “15 + BFA”. *N* = 3 technical replicates. GzmB labelling may be seen in Supplemental Fig. 2C. A *t*-test was used to assess significance, without pairing. An asterisk indicates comparisons which yielded a *p*-value of less than 0.05. **G** T cell viability at baseline and 15-h after seeding on SK37 with or without BFA supplementation. Gates were applied to assay GFP + CD45 + T cells which did not take up viability dye. (H) Bar graph comparing viability of T cells, from (G). *N* = 3 technical replicates. A *t*-test was used to assess significance, without pairing. An asterisk indicates a comparison which yielded a *p*-value less than 0.05

We proceeded to assess the impact of Tcf-1 on the cytolytic activity of CD8^+^ T-cells engineered to express an antitumor TCR derived from tumor-infiltrating-lymphocytes: the 19,305-TCR, one which confers recognition of NY-ESO-1 presented in the context of HLA-A*02:01 [25]. CD8^+^ T cells were engineered to overexpress Tcf-1 and the 19,305-TCR and afterward implemented in co-cultures with SK-MEL-37 melanoma cells (SK37), a cell line that expresses NY-ESO-1 and HLA-A*02:01. Briefly, CD8^+^ T cells were isolated from PBMC and (i) co-transduced to express the 19,305-TCR and Tcf-1 or the 19,305-TCR and GFP (Fig. 3A) (ii) conditioned to advanced cytotoxicity via serial propagation over monolayers of SK37 in media supplemented with IL-2 at 300 IU/ml or 1,500 IU/ml, with passage once every seven days for 21-days (iii) implemented in 24-h co-cultures with SK37 for assessment of cytotoxicity and susceptibility to AICD. Engagements between T-cells and SK37 resulted in substantial reductions in T-cell viability which were mitigated by Tcf-1 (Fig. 3B, C). Tcf1^Tg^ T cells were less cytotoxic than control cells and underwent reduced rates of AICD: Tcf1^Tg^ T cells conditioned with 1500 IU/ml IL-2 were two-thirds as cytotoxic as control cells but retained three-fold greater viability (Fig. 3D, E). We speculated that the observed resistance to AICD could be due to differences in GzmB synthesis in response to conjugation of SK37: that TCR-directed GzmB synthesis could result in increased rates of GzmB leakage into the cytoplasm. Therefore, we assayed GzmB content and viability at baseline and after 15-h of co-culture with SK37. Whereas GFP^Tg^ T cells assayed at baseline had substantial GzmB content and viability, T cells assayed after co-culture contained tenfold greater per cell GzmB levels than cells assayed at baseline and were significantly more apoptotic (Fig. 3F–H). Tcf1^Tg^ CD8^+^ T-cells did not upregulate GzmB as robustly as GFP^Tg^ CD8^+^ T-cells and retained significantly greater viability. We set up co-cultures with Brefeldin-A (BFA) with the expectation that BFA-mediated inhibition of translation would antagonize synthesis of proteins like GzmB [34]. BFA prevented T-cells from upregulating GzmB and acquiring an apoptotic profile (Fig. 3F–H). These results indicate that (i) upregulation occurs as a result of target-cell engagement and increases CD8^+^ T cell susceptibility to AICD (ii) Tcf-1 mediated GzmB restriction limits AICD but does not prevent T cells from engaging in cytotoxic activity, although cytotoxicity is limited.

### Tcf-1 mitigates apoptosis and promotes stem-cell-like cycling activity

**Fig. 4:**
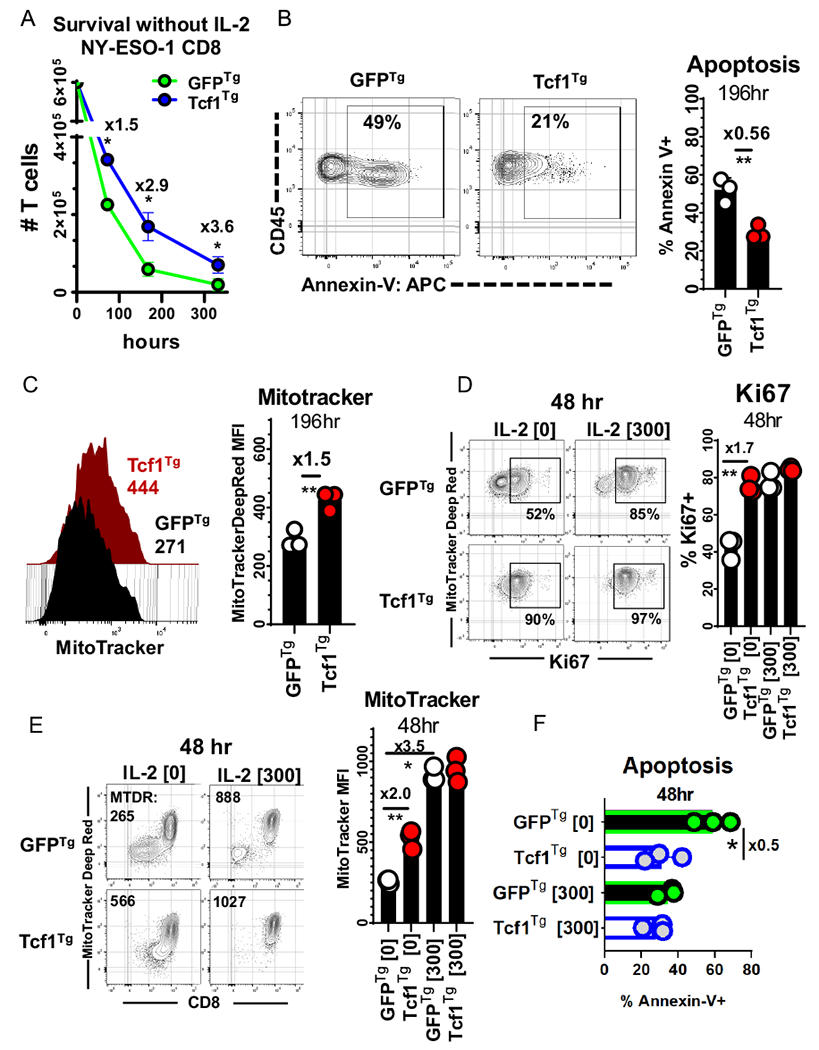
Tcf1^Tg^ TCR-engineered CD8^+^ T cells are reinforced against apoptosis **A** Persistence of TCR-engineered T cells over the course of in vitro culture with SK37 without exogenous IL-2. CD8^+^ T cells were engineered to express the 19,305-TCR and Tcf-1 and were then seeded onto SK37 monolayers without IL-2 supplementation. After (72, 196, 336) hours T cells were collected with uniform technique and were assayed via flow-cytometry-based detection of CD45 + cells with forward-scatter and side-scatter properties typical of lymphocytes, as seen in Supplemental Fig. 3A. CountBrite Absolute Counting Beads were used to ascertain cell numbers. A *t*-test was used to compare cell numbers at each timepoint, with an asterisk indicative of a comparisons that yielded a *p*-value less than 0.05. **B** Viability of TCR-engineered T cells during extended co-culture with SK37. T cells were labelled with annexin-v after 196-h of co-culture. A gate captures cells which labelled positive for annexin-v. CD45 + GFP + Zombie− ells with lymphocyte forward-scatter and side-scatter profiles were assayed. To the right is a bar graph comparing frequencies of apoptotic cells. A *t*-test was applied to assess statistical significance, without pairing. *N* = 3 clones propagated from the same donor. Asterisks indicate comparisons which yielded a *p*-value less than 0.05. **C** Mitochondria polarization of T cells during extended co-culture with SK37, as assessed via labelling with MitoTrackerDeepRedFM [10 nM] after 196-h. To the left are flow-cytometry histograms for T cells transduced to express Tcf-1 (red) or GFP (black) (along with the 19,305-TCR), with median fluorescence intensity listed underneath. Gates (seen in Supplemental Fig. 3B) allowed detection of MitoTracker in viable cells (with CD45 + GFP + Zombie− profiles). To the right is a bar graph. A *t*-test was applied to assess statistical significance, without pairing, with asterisks reflective of comparisons which yielded a *p*-value less than 0.05. *N* = 3 clones from the same donor. **D** Cell-cycle activity of T cells cultured with SK37, as influenced by IL-2. T cells were assayed for Ki67 content 48-h after initiation of co-culture, with or without IL-2 supplemented media (300 IU/ml). Gates capture cells which had intermediate and high labelling for Ki67. CD45 + GFP + CD8^+^ T cells were assayed, as seen in Supplemental Fig. 3C. To the right is a graph comparing frequencies of Ki67 + cells. *t*-tests were applied without pairing, for *N* = 3 clones from the same donor. Asterisks indicate comparisons which yielded a *p*-value less than 0.05. **E** MitoTrackerDeepRedFM labelling [10 nM] in relation to CD8 co-receptor labelling, in T cells co-cultured for 48-h with SK37. Gates were applied to observe CD45 + GFP + cells which did not uptake viability dye. To the right is a bar graph comparing MitoTracker labelling. *t*-tests were used to assess significance, without pairing. *N* = 3 clones from the same donor. Asterisks indicate comparisons which yielded a *p*-value less than 0.05. **F** Viability after 48-h of co-culture. This graph compares the annexin-v labelling of Tcf-1 transgenic TCR-engineered CD8^+^ T cells to GFP transgenic TCR-engineered CD8^+^ T cells. It compares CD45 + GFP + CD8^+^ T cells after 48 h of co-culture with SK37. A *t*-test was used to assess significance, without pairing. *N* = 3 clones from the same donor. Asterisks are indicative of comparisons which yielded a *p*-value of less than 0.05

We used extended co-cultures to compare the survival of Tcf1^Tg^ TCR-engineered CD8^+^ T-cells to that of control cells. Since IL-2 deprivation is a standard test of T cell survival, T cells were cultured without IL-2;[35] equal numbers of CD8^+^ T cells were cultured with SK37 (without IL-2) and counted at concurrent time-points via flow-cytometry, using CD45 alone to identify T-cells (because CD8 surface levels change in relation to activation) [36]. Tcf1^Tg^ T cells were collected in greater numbers at all time points and were more viable than GFP^Tg^ T cells (Fig. 4A, B). We set up additional co-cultures and made multiparameter assessments with cells cultured 48-h with and without support by exogenous IL-2. Mitochondrial polarization was indexed with MitoTrackerDeepRed^FM^(MTDR), via labelling with a 10 nM solution. MTDR-labelled Tcf1^Tg^ T-cells were significantly brighter than controls (Fig. 4C). Ki67 was highly expressed by Tcf1^Tg^ T cells but not GFP^Tg^ T cells. IL-2 allowed control T cells to resemble Tcf1^Tg^ T cells with respect to cell-cycle activity, mitochondria-polarization, and viability (Fig. 4D–F).

**Fig. 5:**
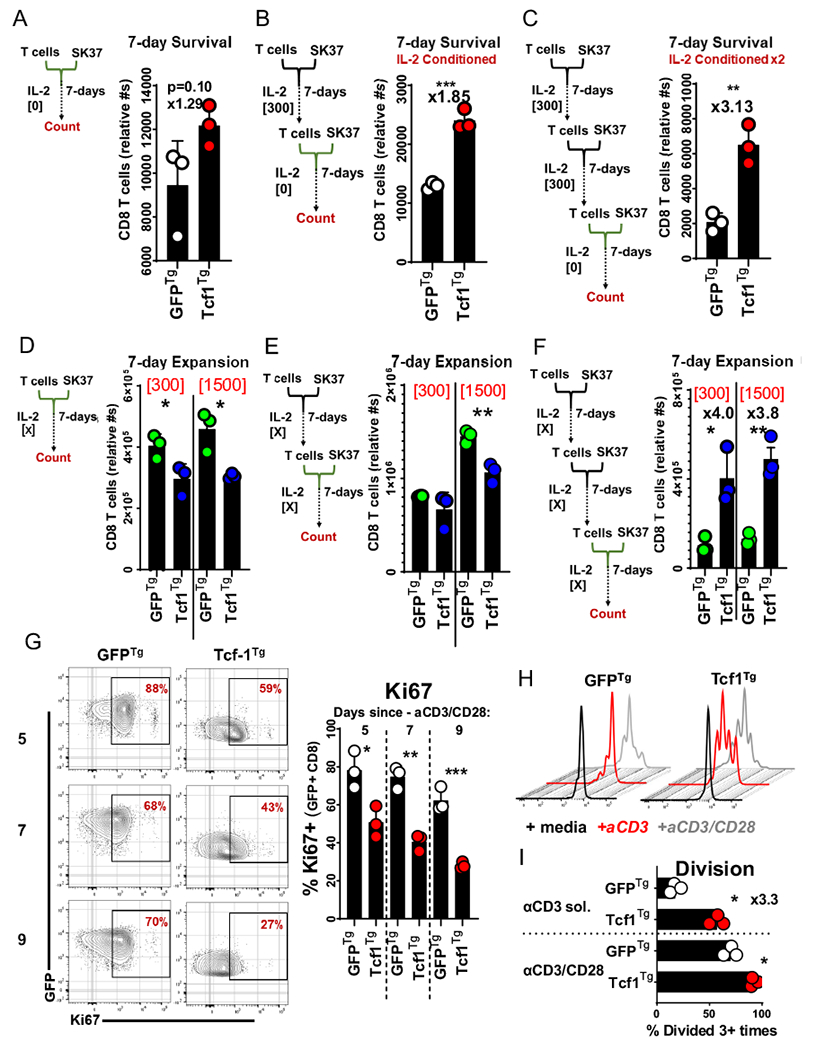
Population dynamics of Tcf1^Tg^ TCR-engineered CD8^+^ T cells **A** CD8^+^ T cell survival of co-culture with SK37 without IL-2, without prior conditioning. 2 × 10^5^ T cells transduced to express the 19,305-TCR and TCF-1B-GFP (or GFP) were seeded into cultures with SK37. After 3-days, media was added. After seven days, T cells were collected and counted via flow-cytometry. T cell numbers were ascertained using CountBrite beads. T cells were discerned from SK37 and beads by forward-scatter and side-scatter properties as well as by CD45 expression. A *t*-test was used to assess statistical significance. *N* = 3 clones derived from the same donor. **B** T cell survival of co-culture with SK37 without IL-2, after conditioning via implementation in a 7-day expansion culture with SK37 and IL-2 supplemented media. 2 × 10^5^ T cells transduced to express the 19,305-TCR and TCF-1B-GFP were seeded into cultures with SK37. After 3-days, media was added. After 7-days, T cells were collected and counted via flow-cytometry. T cell numbers were ascertained using CountBrite beads. T cells were discerned from SK37 and beads by forward-scatter and side-scatter properties as well as by CD45 expression. A *t*-test was used to assess statistical significance, without pairing, and is indicated by asterisks to have yielded a *p*-value below 0.05. *N* = 3 clones derived from the same donor. **C** T cell survival of co-culture with SK37 without IL-2, after conditioning via implementation in serial 7-day expansion cultures with SK37 and IL-2 supplemented media. 2 × 10^5^ T cells transduced to express the 19,305-TCR and TCF-1B-GFP were seeded into cultures with SK37. After 3-days, media was added. After 7-days, T cells were collected and counted via flow-cytometry. T cell numbers were ascertained using CountBrite beads. T cells were discerned from SK37 and beads by forward-scatter and side-scatter properties as well as by CD45 expression. A *t*-test was used to assess statistical significance, without pairing, and is indicated by asterisks to have yielded a *p*-value below 0.05. *N* = 3 clones derived from the same donor. (D) T cell expansion via co-culture with SK37 with IL-2, without prior conditioning. 2 × 10^5^ T cells transduced to express the 19,305-TCR and TCF-1B-GFP (or GFP) were seeded into cultures with SK37, supplemented with IL-2 at 300 or 1500 IU/ml. After 3-days, media was added. After 7-days, T cells were collected and counted via flow-cytometry. T cell numbers were ascertained using CountBrite beads. T cells were discerned from SK37 and beads by forward-scatter and side-scatter properties as well as by CD45 expression. A *t*-test was used to assess statistical significance, without pairing, and is indicated by asterisks to have yielded a *p*-value below 0.05. *N* = 3 clones derived from the same donor. (E) T cell expansion via co-culture with SK37 with IL-2, after conditioning via implementation in a 7-day expansion culture supplemented with an identical concentration of IL-2. 2 × 10^5^ T cells transduced to express the 19,305-TCR and TCF-1B-GFP (or GFP) were seeded into cultures with SK37, supplemented with IL-2 at 300 or 1500 IU/ml. After 3-days, media was added. After 7-days, T cells were collected and counted via flow-cytometry. T cell numbers were ascertained using CountBrite beads. T cells were discerned from SK37 and beads by forward-scatter and side-scatter properties as well as by CD45 expression. A -test was used to assess statistical significance, without pairing, and is indicated by asterisks to have yielded a *p*-value below 0.05. *N* = 3 clones derived from the same donor. (F) T cell expansion via co-culture with SK37 with IL-2, after conditioning via implementation in serial 7-day expansion cultures supplemented with identical concentrations of IL-2. 2 × 10^5^ T cells transduced to express the 19,305-TCR and TCF-1B-GFP were seeded into cultures with SK37, supplemented with IL-2 at 300 or 1500 IU/ml. After 3-days, media was added. After 7-days, T cells were collected and counted via flow-cytometry. T cell numbers were ascertained using CountBrite beads. T cells were discerned from SK37 and beads by forward-scatter and side-scatter properties as well as by CD45 expression. A *t*-test was used to assess statistical significance, without pairing, and is indicated by asterisks to have yielded a *p*-value below 0.05. *N* = 3 clones derived from the same donor. **G** Effect of Tcf-1 on the onset of stasis after cell manufacture, as indexed by Ki67 on days 5–9 after removal of anti-CD3/CD28 dynabeads from cultures. To the left, days are indicated for time since bead removal. PBMC were activated with anti-CD3/CD28 dynabeads and transduced to express GFP or TCF-1B-GFP. After 4-days, T cells were de-adhered from anti-CD3/CD28 dynabeads and seeded for into cultures with IL-2 supplemented media (300 IU/ml). After 9-days, T cells were assayed for the first time point: 5-days after removal of anti-CD3/CD28 dyna-beads. Media was replenished every 48 h. Gates were applied to assess GFP + CD8^+^ T cells. To the right is a bar graph comparing Ki67 positivity. A *t*-test was used to assess significance, with pairing, with asterisks indicative of *p*-values less than 0.05. *N* = 3 donors. (H) Effect of Tcf-1 on cell division elicited after the onset of stasis, following cell manufacture. T cells from (G) were labelled with Cell Trace Violet and re-stimulated with solubilized anti-CD3 antibodies or anti-CD3/CD28 dynabeads in IL-2 supplemented media for five days. Cell division profiles for GFP + CD8^+^ T cells, transduced to express GFP alone or TCF-1B-GFP are shown to the left and right, respectively. Histograms are shown for cells without stimulation (black), anti-CD3 antibodies (red), and anti-CD3/CD28 dynabeads (grey). **I** Effect of Tcf-1 on cell division elicited after the onset of stasis, quantified by bar graphs, as evaluated by the percentage of GFP + CD8^+^ T cells that had undergone three or more divisions. A paired *t*-test was used to assess significance, with pairing, with an asterisk indicative of a comparison which yielded a *p*-value of less than 0.05. *N* = 3 donors

CD8^+^ T cell biomass, viability, and protein content is halved by just 48 h of IL-2 deprivation in vitro [37]. Since TCR or CAR transgenic T cells are typically expanded in IL-2 for 14 days before therapy, there is an opportunity for cells to be conditioned to a state of differentiation more susceptible to apoptosis from IL-2 deprivation, something understood to limit the efficacy of cell-therapy T cells, especially those expanded from tumor-infiltrating-lymphocytes [35, 38, 39]. To examine the effect of Tcf-1 on this process, T cells were (i) conditioned via expansion in SK37 co-cultures with IL-2, (ii) seeded into co-cultures with SK37 without IL-2, and (iii) counted after 7 days via flow-cytometry-based detection of T cells and counting beads. This was done with the expectation that T cells would be subject to a test of survival which would be influenced by prior conditioning. Non-conditioned T cells were collected in similar numbers, although more Tcf1^Tg^ T cells could be collected (Fig. 5A). Tcf1^Tg^ T cells were collected in significantly greater numbers than GFP^Tg^ T cells when implemented after 7 or 14 days of prior conditioning (Fig. 5B, C). To assess the impact of Tcf-1 on expansion, we did similar experiments but with IL-2 support. The addition of IL-2 changes the assay kinetics. When the assay is performed with IL-2, T cell expansion is more pronounced. Further, SK37 monolayers are depleted: IL-2 supports population dynamics and effector functions which allow T cells to clear SK37 and continue to expand. Tcf1^Tg^ T cells underwent limited expansion in these co-cultures when implemented without prior conditioning, and when implemented after just 7 days of conditioning (Fig. 5D, E). However, with 14 days of conditioning, Tcf1^Tg^ cells were collected in four-fold greater numbers (Fig. 5F), likely as a result of their enhanced survival rather than cell division activity.

Since Tcf-1-transgenic T cells from syngeneic models have been reported to incorporate BrdU less readily than wild-type T-cells, [40] we decided to examine the effect of Tcf-1 on cycling activity at a higher resolution. T-cell expansion occurs via proliferative bursts; TCR signaling instructs the duration of time a T-cell and its immediate progeny undergo successive rounds of division before becoming static [41]. Activation states initiated by TCR-signaling are maintained by IL-2 such that T cells can be expanded for extended periods in vitro via aggressive replenishment of IL-2 support. After the cessation of activation, cells return to quiescence, a state understood to leave T cells poised for secondary activation. When this trajectory is studied in vivo in the context of antigen depletion, memory precursors are observed to cycle at reduced rates relative to more differentiated cells. Intrinsic regulation of cell-cycle activity is evidenced by findings that FoxO1 constrains cell activation and could have implications for cell manufacture [9]. Therefore we studied cell-cycle activity in the context of expansion after transduction, when activation states initiated by anti-CD3/CD28 dynabeads are maintained by IL-2. We assayed Ki67 in the final days of a 14-day protocol in which dynabeads are removed after 5-days. GFP^+^ Tcf1^Tg^ T-cells had reduced Ki67 levels relative to GFP^+^ control cells and levels further declined with time (Fig. 5G). When re-stimulated, GFP^+^ Tcf1^Tg^ T-cells divided robustly relative to control cells, and were observed to undergo more rounds of division in the same amount of time (Fig. 5H, I). These results are consistent with a model where Tcf-1 checks cell-cycle activity via a regulatory mechanism that can be overcome by TCR signaling. Since Tcf-1 transgenic PG13 fibroblast cell-lines expand at reduced rates relative to parental cell lines (data not shown), the effect is likely independent of input from IL-2 receptors and T cell receptors.

### Effect of Tcf-1 overexpression on the anti-tumor activity of TCR-engineered CD8^+^ T-cells

**Fig. 6:**
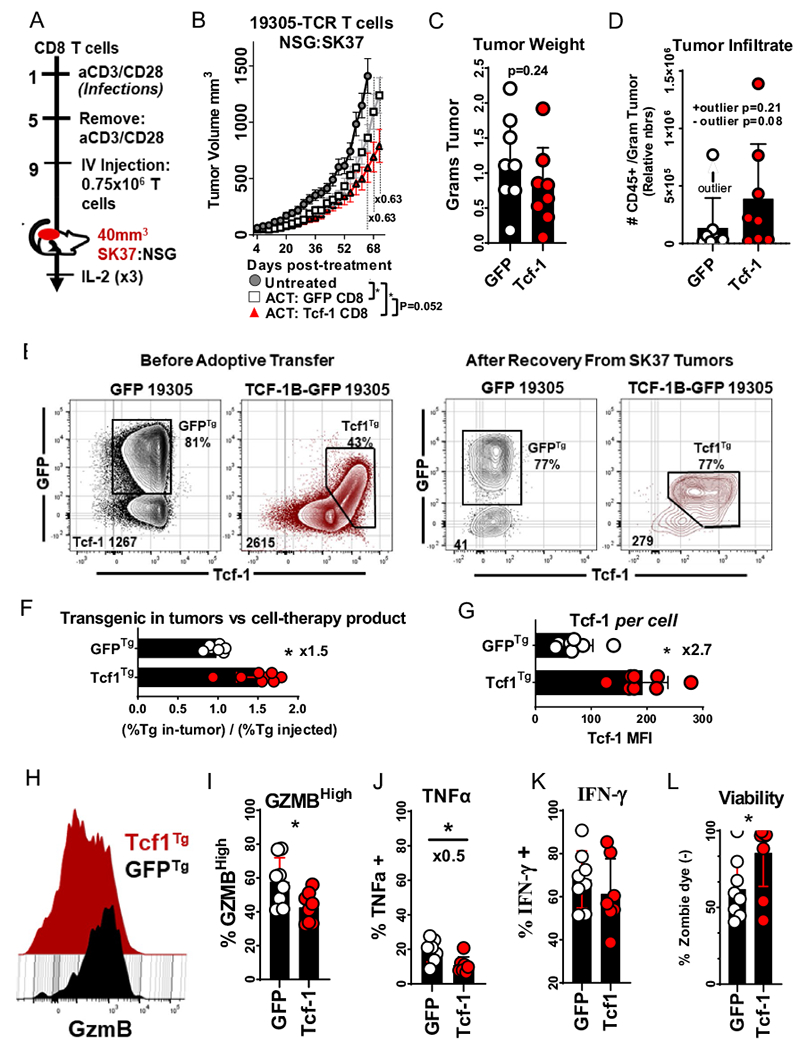
Effect of Tcf-1 on the Anti-tumor activity of a TCR-engineered cell-therapy product **A** Scheme to study the effect of Tcf-1 on the efficacy of 19,305-TCR engineered CD8^+^ T cells, using a xenograft model of adoptive transfer. Human CD8^+^ T cells were (i) purified with Miltenyi CD8^+^ microbeads (ii) activated with anti-CD3/CD28 dynabeads at a 2:3 bead to T cell ratio (iii) transduced to express the NY-ESO-1 specific 19,305-TCR and Tcf-1 (iv) Injected intravenously via the retro-orbital sinus into NSG mice after 9-days of cell manufacture in media supplemented with IL-2 (300 IU/ml). (0.75 × 10^6^) CD8^+^ T cells were injected into NSG mice with 40mm^3^ SK37 tumors. Mice were injected with IL-2 in the peritoneum on the day of injection and every 24-h for 72 h, (3 × 50,000 IU Goldbio IL-2). **B** Effect of Tcf-1 on efficacy of cell-therapy CD8^+^ T cell product, as indexed by measurement of subcutaneous SK37 melanoma tumors. Mice with well-established tumors of 40mm^3^ were split into three treatment groups, (i) those which received CD8^+^ T cells transduced to express TCF-1B-GFP and the 19,305-TCR (Red) (ii) those which received CD8^+^ T cells transduced to express GFP and the 19,305-TCR (white) (iii) those that did not receive treatment (gray). Volumes were determined via the modified ellipse formula V = (L × W^2^)/2. Tumors were measured every 72-h. Superimposed lines allow comparison of mice treated with Tcf-1 transgenic T cells to mice treated with control T cells, with tumors reaching volumes ~ 2/3 (× 0.63) of that of controls. Statistical significance was assessed using *t*-tests, which were completed without pairing. Significance is assigned below the plot, with asterisks indicative of a *p*-value of less than 0.05. Error bars represent one standard error about the mean (SEM). *N* = 9 mice. **C** Weight of SK37 tumors resected from mice at the end of the study. Statistical significance was assessed with a *t*-test, without pairing. *N* = 8 mice **D** Influence of Tcf-1 on tumor-infiltration by TCR-engineered CD8^+^ T cells. Tumors were minced and enzymatically digested into single cell suspensions. An equal proportion of each suspension was spiked with CountBrite beads and assayed for CD45 + T cells using flow-cytometry. The gating strategy to count CD45 + T cells is found in Supplemental Fig. 4B. The algorithms used to calculate cell numbers per gram of tumor are found in the Supplemental Methods. A *t*-test was used to assess statistical significance, without pairing. *N* = 8 tumors. The TCR content of the cells used is available in Supplemental Fig. 4a. *E* Characterization of the Tcf-1 transgenic cell-therapy product before injection and after recovery from tumors; CD8^+^ T cells were assayed for intracellular Tcf-1 content prior to implementation in ACT, and after recovery from tumors. To the left is a set of plots depicting the Tcf-1 content of cells briefly stimulated with mitogens to facilitate maximal detection of Tcf-1 and GFP. To the right is a set of plots depicting cells recovered from tumors, acquired per the gating strategy seen in Supplemental Fig. 4B. Cells assayed before and after transfer were collected with different cytometry settings and should not be compared. Gates are representative and depict the strategy used to identify transgenic cells based on expression of Tcf-1 and GFP, with the rationale that cells with artificially high Tcf-1 content that express GFP are transgenic. The number in the bottom left corner of each flow plot indicates the median fluorescence intensity of Tcf-1 for the entirety of cells in the plot. The TCR expression of each cell-therapy product is available in Supplemental Fig. 4A. **F** Characterization of the degree to which the Tcf-1 enforced cell-therapy product is enriched for transgenic CD8^+^ T cells after recovery from tumors. Since Tcf-1 transgenic T cells were observed to be at increased frequencies in tumor-infiltrate relative to the pre-injection product, we compared this enrichment to that of GFP control cells which had similar frequencies of GFP positive cells before and after implementation in the model. This assessment is not intended to communicate differences in persistence but rather an observation which may relate to persistence, or which may be technical in nature. Statistical significance was assessed via a *t*-test, without pairing, with an asterisk indicative of a *p*-value of less than 0.05, *N* = 8. **G** Degree to which Tcf-1 is expressed by tumor-infiltrate. The median fluorescence intensity of Tcf-1 was assessed from populations observed in E, to the right. Statistical significance was assessed with *t*-test, without pairing, with asterisks indicative of a *p*-value of less than 0.05, *N* = 8. **H** Effect of Tcf-1 on GzmB expression of tumor-infiltrating TCR-engineered CD8^+^ T cells. T cells were assayed via flow cytometry using the gating strategy seen in Supplemental Fig. 4. **I** Effect of Tcf-1 on GzmB expression, compared via a bar graph. T cells with peak expression of GzmB were captured in a gate which may be viewed in Supplemental Fig. 4C. A *t*-test was used to assess statistical significance, with asterisks indicative of *p*-value of less than 0.05. This test was done without pairing. *N* = 8. **J** Effect of Tcf-1 on the ability of tumor-infiltrating CD8^+^ T cells to produce TNFα in response to stimulation with mitogens and BFA. The cytometry plots for this graph may be viewed in Supplemental Fig. 4C. Statistical significance was assessed with a *t*-test, without pairing. An statistical outlier was identified and removed from the GFP dataset, and the *p*-value would be 0.11 with this outlier included. Sample sizes were *N* = 7 and *N* = 7; one sample from the Tcf-1 group did not yield sufficient numbers of cells for implementation in this assay. (H) Effect of Tcf-1 on the ability of tumor-infiltrating T cells to produce IFN-γ after re-stimulation with mitogens and BFA. The flow-cytometry plots for this quantification are viewed in Supplemental Fig. 4. Statistical significance was assessed with a *t*-test, without pairing. Sample sizes were *N* = 8 and *N* = 7: one sample from the Tcf-1 group did not yield sufficient cells for implementation in this assay. **I** Effect of Tcf-1 on the viability of T cells recovered from tumors, as determined by uptake of Zombie UV viability dye. Flow-cytometry plots may be found in Supplemental Fig. 4C, bottom left. Statistical significance was assessed with *t*-test, without pairing, with an asterisk indicative of a *p*-value of less than 0.05. *N* = 8 samples

We carried out a xenograft model of adoptive cell transfer for the purpose of assessing the influence of Tcf-1 overexpression on the efficacy of TCR-engineered cell-therapy products (Fig. 6A): NSG mice with subcutaneous SK37 melanoma tumors were intravenously injected with CD8^+^ T cells transduced to express Tcf-1 and the 19,305-TCR. This cell-therapy product had identical activity as the control product (GFP and 19,305-TCR transduced) despite it containing an artificial memory compartment composed of Tcf1^Tg^ cells with limited cytotoxicity (Fig. 6B–D). Whereas 43% of cells from the Tcf1^Tg^ cell product were GFP^+^, GFP was expressed by the majority of cells recovered from tumors, for reasons that remain unclear but correlate with expression of Tcf-1. T cells recovered from the tumors of animals injected with Tcf1^Tg^ T cells expressed Tcf-1 at 2.7-fold greater levels than GFP^Tg^ T cells (Fig. 6E–G). Tcf-1 checked T-cell effector function; Tcf-1 (i) prevented T cells from acquiring a GzmB^high^ phenotype (ii) checked TNF-α production in re-stimulated cells (iii) did not have a significant effect on IFN-γ production (Fig. 6H–L). These results indicate that the inclusion of a Tcf-1 enforced memory compartment does not hamper the anti-tumor activity of TCR-engineered CD8^+^ T-cells.

## Discussion

Since Tcf-1 is the prototypical identity factor for potent immunotherapy CD8^+^ T cells with attributes of stemness [1, 10, 40], we interrogated its ability to promote stem-cell-like functions and persistence in TCR-engineered T-cells. Tcf-1 promoted functions (i) that have the potential to synergize with programs imparted on cell-therapy T cells (ii) which provide understanding as to the mechanisms by which stemcell-like T-cells persist. Naïve T cells express Tcf-1 at peak levels and persist as quiescent cells which have yet to acquire expression of granzymes, effector cytokines, or IL-2. The Tcf-1^+^ memory T-cells they give rise to are often described as having optimal potential for cell-therapy because of their intrinsic longevity [1]. The mechanisms by which these cells persist remain elusive as do strategies to manufacture therapeutic T cells with memory attributes. Here, we reduced cell persistence to survival of discrete engagements with cancer cells and observed T cells to undergo apoptosis as a result of the elicitation of their cytotoxicity. We found that the main function of Tcf-1 is to protect T-cells from apoptosis and that this occurs via regulation of GzmB. We also found evidence of regulation of GzmB expression in post-naïve stages of differentiation where Tcf-1 is abundantly expressed, consistent with findings by other groups [20, 29]. Accordingly, we conclude GzmB to be an intrinsic checkpoint on persistence that contributes to the fragility of effector cells. This is consistent with previous reports in murine T cells indicating that GzmB is expressed at notably lower levels by T-cells imparted with genetic enhancement of Tcf-1 or upstream-regulator FoxO1 [9].

While cytotoxic lymphocytes are capable of tumor control in the absence of GzmB, [17, 42] the GzmB-exocytosis pathway is significant for cytotoxic function. Therefore, Tcf-1 overexpression presents a tradeoff. This tradeoff may or may not influence ACT-performance, depending on the ability of the cells to utilize non-granzyme centered pathways against tumor cells. The effect of Tcf-1 on in vivo IFN-γ producing capabilities was insignificant, and limited in magnitude relative to its effect on GzmB. Tcf-1-engineered cells may primarily enact anti-tumor activity via IFN-γ, TNF-α, and FAS-ligand. In line with this, CD8^+^ T-cells from syngeneic mice containing constitutive Tcf-1 transgenes were recently reported to have potent anti-tumor activity because of their ability to persist and retain polyfunctional cytokine production [40]. Here, we report a proof-of-concept approach to applying constitutive Tcf-1 expression to TCR-engineered human T cells. Differences in the results obtained between our study and the one discussed may be attributed to the methodologies used; constitutive Tcf-1 expression would ideally be studied in the context of intact and immunogenic tumor microenvironments.

Tumor-infiltrating lymphocytes (TIL) are typically effector cells with peak GzmB expression. TIL are susceptible to attrition because of their extended ex-vivo preparation with anti-CD3 antibodies and IL-2 [43]. Their use is somewhat limited by their requirement for exogenous IL-2 and susceptibility to apoptosis in its absence [35]. Signals transmitted by IL-2 receptors maintain anti-apoptotic Bcl-2 proteins and ultimately antagonize mitochondria permeabilization and cytochrome-C release, the dominant axis of GzmB-mediated apoptosis [19]. Since Tcf-1-transduced T-cells had a modest in vitro survival advantage in the absence of IL-2, and this advantage was potentiated by conditioning that made T cells more cytotoxic, it is possible that the limited GzmB content of Tcf1^Tg^ T cells could translate to a decreased reliance on IL-2 for maintenance of mitochondrial-integrity and survival. Such would assume the existence of a stoichiometric tug-of-war at mitochondria between IL-2-supported anti-apoptotic Bcl-2 proteins and GzmB-activated pro-apoptotic BID. This mechanism could explain the elevated IL-2 requirements of effector cells which generally express GzmB at peak levels and would be consistent with a model where IL-2 exists primarily to reinforce mitochondrial stability in Tcf-1 deficient stages of differentiation where T-cells have peak GzmB expression. In the context of the resolution of an endogenous immune response, this axis would allow the selective deletion of effector cells (over GzmB-low memory cells) when IL-2 levels decline.

An unresolved question in CD8^+^ T-cell biology is whether the tendency of Tcf-1 expressing T-cells toward quiescence is programmed or rather simply results from depletion of signals which maintain a heightened state of activation. We observed evidence of Tcf-1 mediated checks on cell cycle activity consistent with an entry into quiescence, as has been reported by others, and which is strikingly similar to effects mediated by FoxO1, the upstream regulator of Tcf-1 [9]. We propose that Tcf-1 promotes cell-cycle egress via a modest effect that is penetrant when cycling activity initiated by TCR-signaling is maintained by IL-2. IL-2 driven cell-cycle activity promotes terminal differentiation, and synthetic alternatives to IL-2 which are less expansive are touted for their potential for manufacture of therapeutic T-cells with increased in vivo activity [44].

There are potential limitations to pursuing methods to engineer persistent T cells. A principal concern is whether durable T cell responses could perpetuate off-tumor on-target toxicity. Transferred T cells may theoretically contain endogenous TCRs capable of self-recognition. Transferred cells could elicit pathologies via endogenous TCRs or alternatively perpetuate underlying T cell responses against self-antigen via secreted cytokines. Although the preclinical model studied here was inadequate to address these issues, each should be considered in the context of the specificity of the TCR or CAR. In this regard, the specificity of the NY-ESO-1 TCR utilized in our studies has been demonstrated by extensive testing for cross-reactivity to HLA-A*02:01^+^ and non-HLA-A*02:01^+^ targets pulsed with NY-ESO-1 or irrelevant peptides [25]. While insertional oncogenesis represents another potential concern which can occur in gene-modified cells, [45] these events are relatively rare in T cells modified using gamma retrovirus [46]. To address these potential limitations, constructs delivered to engineered T cells could be addressed via suicide genes which instigate apoptosis in response to administered agents [47].

In conclusion, within the context of cell-manufacture protocols in which cells are expanded in vitro after engineering, Tcf-1 mediated checks on post-activation cycling activity could provide extended instructions to divide, and limit GrzB mediated self-destruction. In the context of an endogenous immune response, a return to quiescence after antigen-experience could allow cells to engage in surveillance activity, mitigate bioenergetic requirements associated with excessive activation, and resist stress from DNA-damage associated with cell division.

## Supplementary Material

1817327-Sup_Material

## Data Availability

The datasets used in this manuscript are available upon request.

## Abbreviations

AICD: Activation-induced-cell-death
dnTCF-1B: B isoform of Tcf-1 lacking trans-activation domains, beta-catenin domain
GzmB: Granzyme-B
PBMC: Peripheral blood mononuclear cells, collected from leukoreduction chambers
Tcf-1: The T Cell Factor One (TCF-1) gene 5Q31.1 and protein
TCF-1B: B isoform of Tcf-1
TCF-1E: E isoform of Tcf-1
Tcf1^Tg^: Tcf-1 transgenic, cells infected to express the TCF-1B-GFP transgene
TCR: T cell receptor
