# Correction to: Tcf‑1 protects anti‑tumor TCR‑engineered CD8^+^ T‑cells from GzmB mediated self‑destruction

**DOI:** 10.1007/s00262-022-03233-1

**Published:** 2022-06-06

**Authors:** Brendan Zangari, Takemasa Tsuji, Junko Matsuzaki, Hemn Mohammadpour, Cheryl Eppolito, Sebastiano Battaglia, Fumito Ito, Thinle Chodon, Richard Koya, A. J. Robert McGray, Kunle Odunsi

**Affiliations:** 1 Department of Immunology, Roswell Park Comprehensive Cancer Center, Buffalo, NY USA; 2 University of Chicago Medicine Comprehensive Cancer Center, 5841 S Maryland Ave, Chicago, IL 60637 USA; 3 Department of Obstetrics and Gynecology, University of Chicago, Chicago, IL USA; 4 Department of Genetics and Genomics and Bioinformatics, Roswell Park Comprehensive Cancer Center, Buffalo, NY USA; 5 Keck School of Medicine, University of Southern California, Los Angeles, CA USA

## Correction to: Cancer Immunology, Immunotherapy https://doi.org/10.1007/s00262-022-03197-2

The article Tcf‑1 protects anti‑tumor TCR‑engineered CD8+ T‑cells from GzmB mediated self‑destruction, written by Brendan Zangari, Takemasa Tsuji, Junko Matsuzaki, Hemn Mohammadpour, Cheryl Eppolito, Sebastiano Battaglia, Fumito Ito, Thinle Chodon, Richard Koya, A. J. Robert McGray and Kunle Odunsi was originally published electronically on the publisher’s internet portal on 23 April 2022 without open access. With the author(s)’ decision to opt for Open Choice the copyright of the article changed on 25 May 2022 to © The Author(s) 2022 and this article is licensed under a Creative Commons Attribution 4.0 International License, which permits use, sharing, adaptation, distribution and reproduction in any medium or format, as long as you give appropriate credit to the original author(s) and the source, provide a link to the Creative Commons licence, and indicate if changes were made. The images or other third party material in this article are included in the article's Creative Commons licence, unless indicated otherwise in a credit line to the material. If material is not included in the article's Creative Commons licence and your intended use is not permitted by statutory regulation or exceeds the permitted use, you will need to obtain permission directly from the copyright holder. To view a copy of this licence, visithttp://creativecommons.org/licenses/by/4.0/

The original article has been corrected.

