# Supplementary material for: Tcf-1 protects anti-tumor TCR-engineered CD8+ T-cells from GzmB mediated self-destruction
Source: Cancer Immunol Immunother. 2022 Apr 23;71(12):2881–98. doi: 10.1007/s00262-022-03197-2 (PMC9588092; doi:10.1007/s00262-022-03197-2)

Supplemental Materials and Methods

Quantitative PCR Reactions

Reactions:
TCF-1B: 350nM primers. Temperature for optimal annealing: 51.5°C
Exon 6 FWD: CTGCCATCAACCAGATCCTG
Exon 8:10 REV: TTTCCTCCTGTGGTGGATTCTT

TCF-1E: 175nM primers. Temperature for optimal annealing: 60.6 °C
Exon 6 FWD: CTGCCATCAACCAGATCCTG
Exon 9:10 REV: CTTTTTCCTCCTGCACGG

RPL4: 700nM primers. Temperature for optimal annealing: 51.5°C
FWD: CAAGAGTAACTACAACCTTC
REV: GAACTCTACGATGAATCTTC

Cloning Tcf-1 transgenes (sequence information available at the bottom of the methods section)

Custom DNA oligonucleotides specific to regions of interest were synthesized by Integrated DNA Technologies. cDNA from Jurkat cells was used as a template to amplify sequences for assembly, using Q5 High Fidelity Polymerase (New England Biolabs M0491S). PCR products were subjected to electrophoretic mobility shift assays, agarose (1-3%). Products of desired size were excised and stored overnight at 4 ºC in agarose dissolving buffer and extracted with the Monarch DNA Gel Extraction Kit (New England Biolabs T1020). Purified amplicons were assembled into transgenes and integrated into NOT1/PAC1 digested PDONII plasmids with The HiFi DNA Assembly Kit (New England Biolabs E5520S). HiFi assembly reactions were used to transform competent cells ,42 ºC for 30 seconds. Transformants were plated on ampicillin-supplemented agar dishes overnight, and individual colonies were screened for transgene content via Sanger Sequencing. Individual transformants were expanded overnight in LB broth. We purified recombinant pDONII plasmids with the ZymopureII Plasmid Midiprep Kit (ZymoResearch D4200) for transfections.

Preparation of retrovirus coated plates

In order to prepare retrovirus for transductions, oligoclonal PG13 virus producing cells were plated in the 6-well format, at one million cells per well in 2.7mL complete DMEM media. Media was changed 24-hours after plating. After 48-hours, on the day of infection/transduction, supernatant was removed and filtered with a 100uM filter into a 50mL conical, (and was further filtered with a 0.4μM filter). In experiments where GFP

19305-TCR transgenic and Tcf-1 19305-TCR transgenic T cells were prepared, supernatant was mixed at a 1:1 ratio following collection (ratios with a 1:2 19305:GFP ratio have been equally efficient). Supernatant was passaged through a 0.40uM filter and applied to untreated 6-well plates that had been (i) coated with 0.8-1mL of Retronectin diluted in PBS to 10ng/mL for incubation overnight at 4°C (Takara Clontech T100A) (ii) blocked with BSA for 30 minutes. Prior to application to plates, supernatant was concentrated with Retro-X Concentrator (Takara Clontech 631455). Viral pellets were resuspended with pre-warmed T cell media and retroviral suspensions were applied to plates for 3-6 hours. Re-suspended virus was passaged over each well serially with care to ensure Tcf-1 transgenic and control cells were exposed to plates treated identically.

Extraction of tumor infiltrating lymphocytes

Tumors were excised and quickly minced into 5mL ice cold HBSS in a petri dish on ice. Mixtures were brought to 10mL by addition of 5mL of a pre-warmed solution composed of 1mL of 10x Double Enzymatic Tumor Digestion Buffer and 4mL of HBSS. Mixtures were shaken at 225 rpm for 1 hour at 37 ºC in 50mL conical tubes at a 35-degree angle. Mixtures were passaged through 100uM strainers to remove aggregates.

10X Double Enzymatic Tumor Digestion Buffer

HBSS 50mL

Amphoptericin B 5mL@250ug/mL

Collagenase type 4 0.5mg (sigma c5138-500mg)

DNAse 1 (type4) 10,000 units, 5.3mg (sigma D5025-15ku)

Pen/strep 5mL@ 100X

Assaying T cell infiltrate

Tumors were excised and weighed immediately. Single cell suspensions were made with uniform methods to ensure identical volumes were maintained throughout preparation. 3/20th of the total suspension was labelled and assayed on the cytometer for a set period of time to control for volume assayed. 25,000 CountBrite beads were added to each sample immediately prior to cytometry and samples were vortexed. CD45+ cell numbers were counted and compared to bead numbers. We devised an algorithm which would allow us to assess

infiltrate. We reasoned that by controlling the proportion of the single cell suspension (and thus tumor) assayed, differences in the representation of T cells within infiltrate would be apparent via addition of counting standards. We normalized numbers relative to the proportion of the cytometry sample assayed on the cytometer. We normalized by weight to estimate numbers per gram of tumor.

CD45+/Gram tumor=(total CD45+ in 20mL single cell suspension)/grams tumor

(# in 20mL single cell suspension)=(#CD45 counted from 3mL)/(fraction of tube assayed)

Fraction of tube assayed=(beads counted)/25,000

Complete media Reagents

FBS.(Corning 35-011-CF Lot 18120001)

RPMI 1540 with L glutamine. (Corning 10-040-CV)

DMEM 1x with 4.5g/L glucose, L glutamine and sodium pyruvate. (Corning 10-013-CV)

L glutamine 200mM. (Corning 25-005-CL)

MEM nonessential amino acids 100x solution (Corning 25-025-CL)

Hepes Buffer. 1M (Corning 25-060-CL)

Sodium Pyruvate 100mM. (Corning 25-000-CL)

Pen/Strep 100x. (Corning 30-002-CL)

Trypsin 0.25%, 2.21mM EDTA . (Corning 25-053-CL)

Beta-mercaptoethanol

Flow Cytometry

Most experiments used a standard protocol in which surface markers were labelled at 4 ºC for 30 minutes in PBS supplemented with FBS 10%. In experiments which detected intracellular epitopes, the True Nuclear Transcription Factor Buffer Set was used to fix cells overnight at 4 ºC, and permeabilize cells at room temperature for no less than 45 minutes. Intracellular labelling was performed for 45-60 minutes at room temperature. Samples were assayed on LSR-II and Fortessa cytometers (BD Bioscience). Cytometry data was

assessed using Flowjo version 10. Representative gates were superimposed on flow plots to increase their visibility.

Antibodies and reagents for Flow Cytometry

Tcf-1:Ax647 C63D9 Cell Signaling Technologies #6709

Tcf-1:PE C63D9 Cell Signaling Technologies #14456

CD62L:bv650 Biolgend 304832

CD8:bv421 Biolegend 301306

CD3:bv605 Biolegend 317322

IL-2:APC Biolegend 500309

CD28:APC Biolegend 302911

BV Buffer BD Bioscience# 563794

CD8:PE Biolegend 301008

CXCR3:Bv785 Biolegend 353738

IFNγ:PE Biolegend 502509

CD28: Bv785 Biolegend 302349

CD57:APC Biolegend 393305

Ki67:bv605 Biolegend 530522

Ki67: bv421 Biolegend 350505

CD45:PE Biolegend 304008

KLRG1:PercpCy5.5 Biolegend 368611

TNFa:PercpCy5.5 Biolegend 502925

Zombie UV fixable viability kit Biolegend 423107

Annexin-v:APC Biolegend 640919

Live/Dead fixable Aqua. ThermoFisher Scientific #L34957

Mitotracker Deep Red FM. ThermoFisher Scientific M22426

CD57:FITC Biolegend 322306

GzmB:Ax700 BD Bioscience 560213

Vbeta8 TCR:APC Biolegend 348106

Supplemental Figure Legends

Supplemental Figure 1: Dynamic Regulation of Tcf-1 and isoform effects on functionality

(A) Gating strategy to assay Tcf-1 and GzmB expression in CD8 T cells of PMBC, by gating on single cells, lymphocytes, and CD3+ CD8+ CD8 T cells.

(B) Characterization of the qPCR reactions developed to assay the abundance of transcripts coding for carboxylic-terminal isoforms. We focused on TCF-1E and TCF-1B because these are the most abundant isoforms, as described in described in Marc Von Wetering and Han's Clevers 1994 manuscript. E-isoforms and B-isoforms contain exon 8:9:10 and exon 8:10 splice patterns, respectively; The splicing of exon 8 to the 3'-splice acceptor in exon 10 is characteristic of TCF-1B. The splicing of exon 8 to exon 9 and exon 9 to the 3'-splice acceptor in exon 10 results in an exon 10 sequence which differs in identity from that of the B isoform, and which is also longer and characteristic of E-isoforms.

(C) IFNγ production by GFP+ CD8 T cells among PBMC transduced to express GFP or TCF-1-GFP transgenes after re-stimulation. PBMC were transduced, briefly expanded, and further expanded with OKT3 and IL-2 prior to implementation in this assay.

(D) IL-2 production by GFP+ CD8 T cells upon re-stimulation with the Biolegend Cell Activation Cocktail with BFA. PBMC were expanded with OKT3 and IL-2 prior to implementation in this assay.

(E) Figure 2 populations assayed for CD28 and GzmB content after re-stimulation induced expansion; Gating strategy to phenotype GFP+ CD8 T cells which had divided more than 4 times. Gates are applied to $Tcf1^{Tg}$ T cells which had peak Tcf-1 content "Generation 4+ Tcf-1 high", described as $Tcf1^{Tg:High}$ in the text. GFP^{Tg} and $Tcf1^{Tg}$ cells were manufactured from purified naïve human CD8+ T cells. Cells were labelled with CellTrace Violet division dye and expanded with soluble OKT3 (50ng/mL) and IL-2 (300IU/mL) for 5 days. To the right is a bar graph quantifying per cell Tcf-1 content via median fluorescence intensity.

(F) CXCR3 content of GFP+ CD8 T cells in PBMC expanded via standard protocols

Supplemental Figure 2: Cytotoxicity assays and GzmB upregulation

(A) Gating strategy to assay the viability of Tcf1^{Tg} 19305-TCR transduced CD8 T cells and SK37 after 24-hours of co-culture. Forward-scatter and side-scatter were used to discern lymphocytes from SK37. CD45 positivity was assigned with a threshold defined by GFP expression.

(B) Gating strategy to assay the viability of T cells after 15-hour co-culture, by gating on GFP+ CD45+ cells that did not uptake viability dye

(C) GzmB content of T cells at zero hour or after 15 hours of co-culture with SK37, with or without BFA.

Supplemental Figure 3: Population-Kinetics assays

(A) Gating strategy to determine T cell numbers with CountBrite beads. Beads and lymphocytes are stratified by FSC-A / SSC-A. Singlets are stratified into CD45+ and CD45- populations. The CD45+ population is counted. The rightmost plot shows beads, on the axis, as is expected as a result of their characteristic sensitivity to excitation by most lasers. Bead numbers from this gate were input into algorithms to assess cell numbers.

(B) Gating strategy to assay MTDR labelling. Gates were applied to study CD45+ GFP+ Zombie- cells

(C) Gating strategy to assay Ki67 content. Gates are applied to study CD45+ GFP+ cells

Supplemental Figure 4: Characterization of cells used in model of adoptive transfer

(A) CD8 purity and TCR content of cells used in ACT, characterized in **Figure 3A**.

(B) Gating strategy to identify and gate on tumor infiltrating T cells: CD45+ CD8 T cells

(C) Demonstration of the labelling used to assign phenotypes to CD45+ tumor-infiltrating lymphocytes. Cells assayed for cytokine content were re-stimulated prior to cytometry.

Supplemental Figure 1: Dynamic regulation of Tcf-1 & isoform effects on function

A: Gating on CD8 T cells

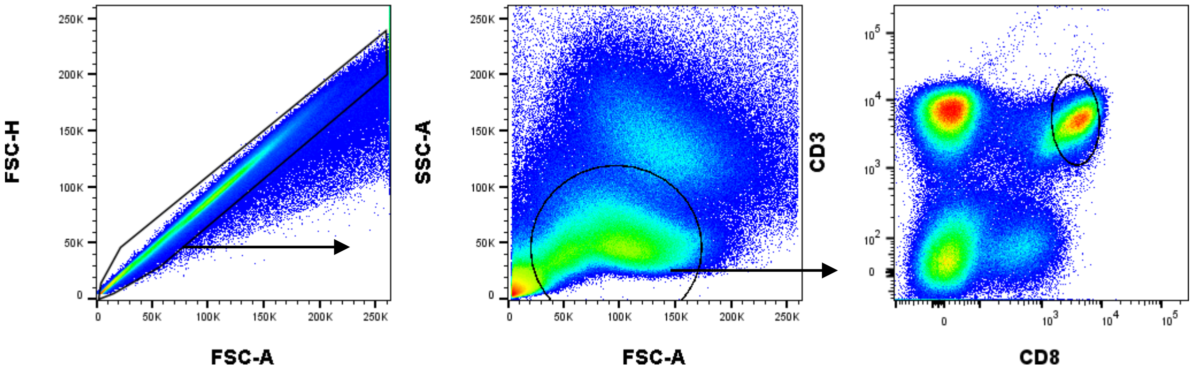

B: Characterization of qPCR assay used to detect TCF-1 splice isoform transcripts

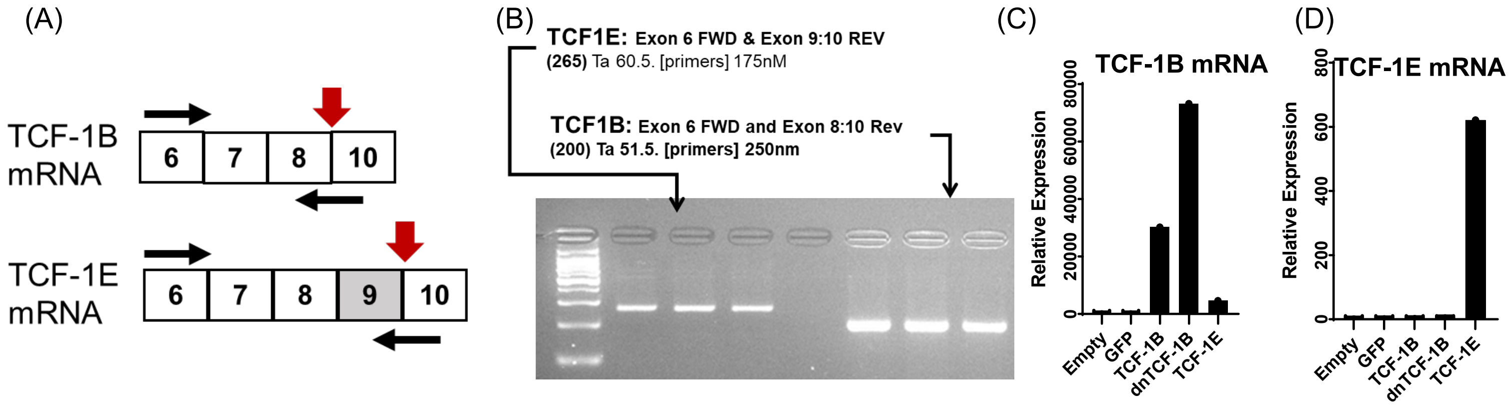

C: IFN-γ production by GFP+ CD8+ T cells in PBMC after restimulation

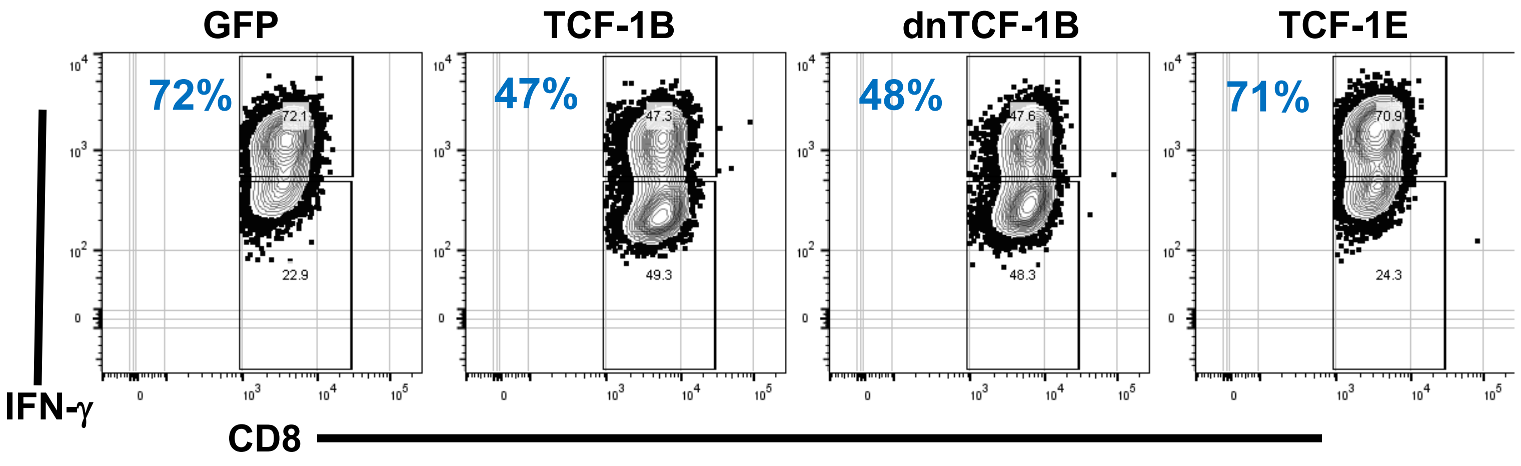

Supplemental Figure 1, page two

D: IL-2 production by GFP+ CD8 T cells

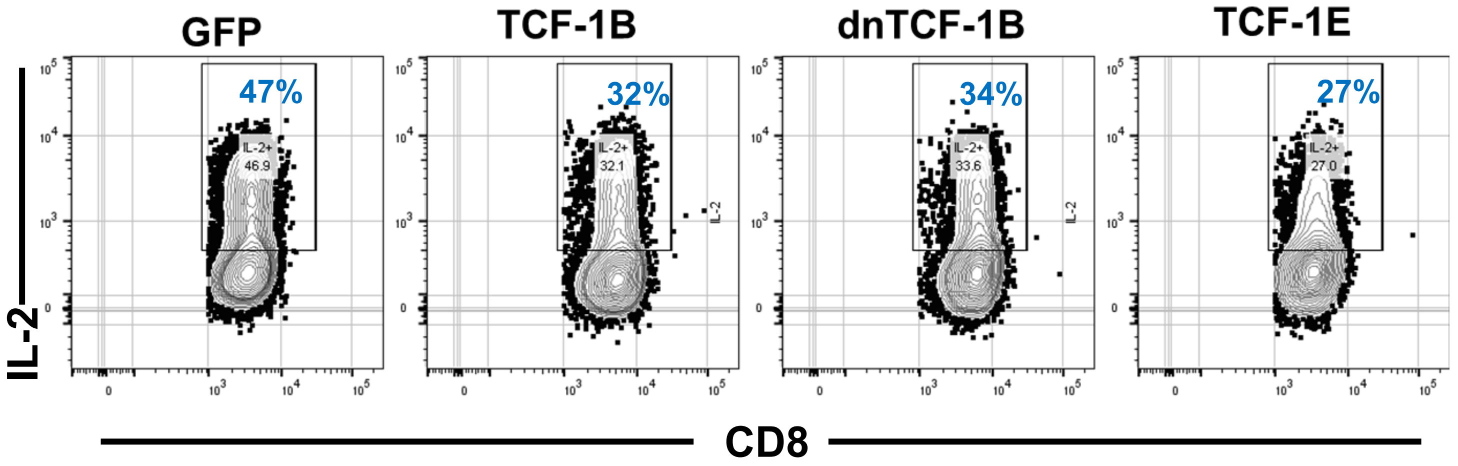

E: Tcf-1 content and division history of cells assayed for CD28 and GzmB content

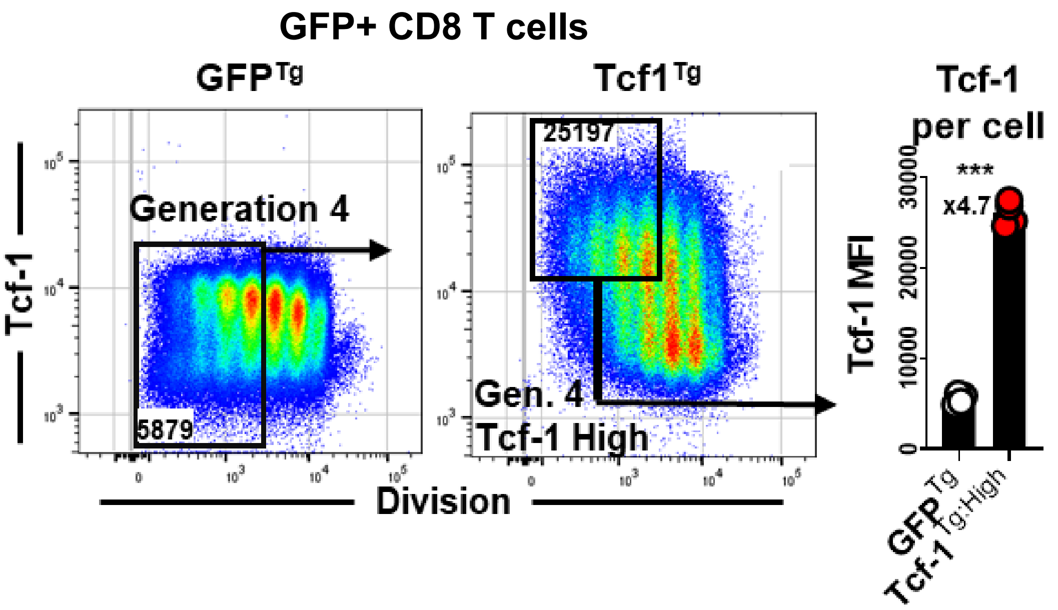

F: CXCR3 content of GFP+ CD8 T cells among PBMC expanded in IL-2 for 9 days

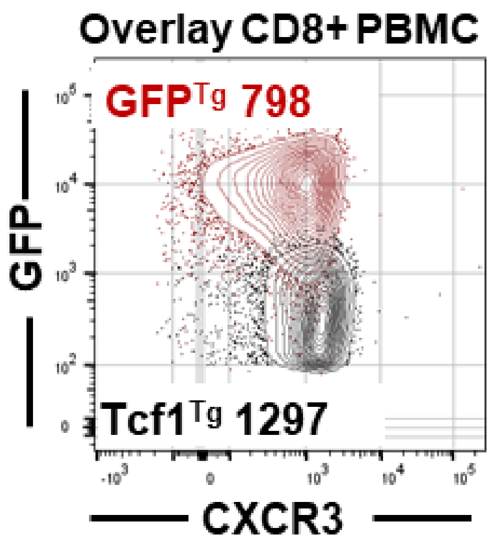

Supplemental Figure 2: Cytotoxicity assays and GzmB upregulation

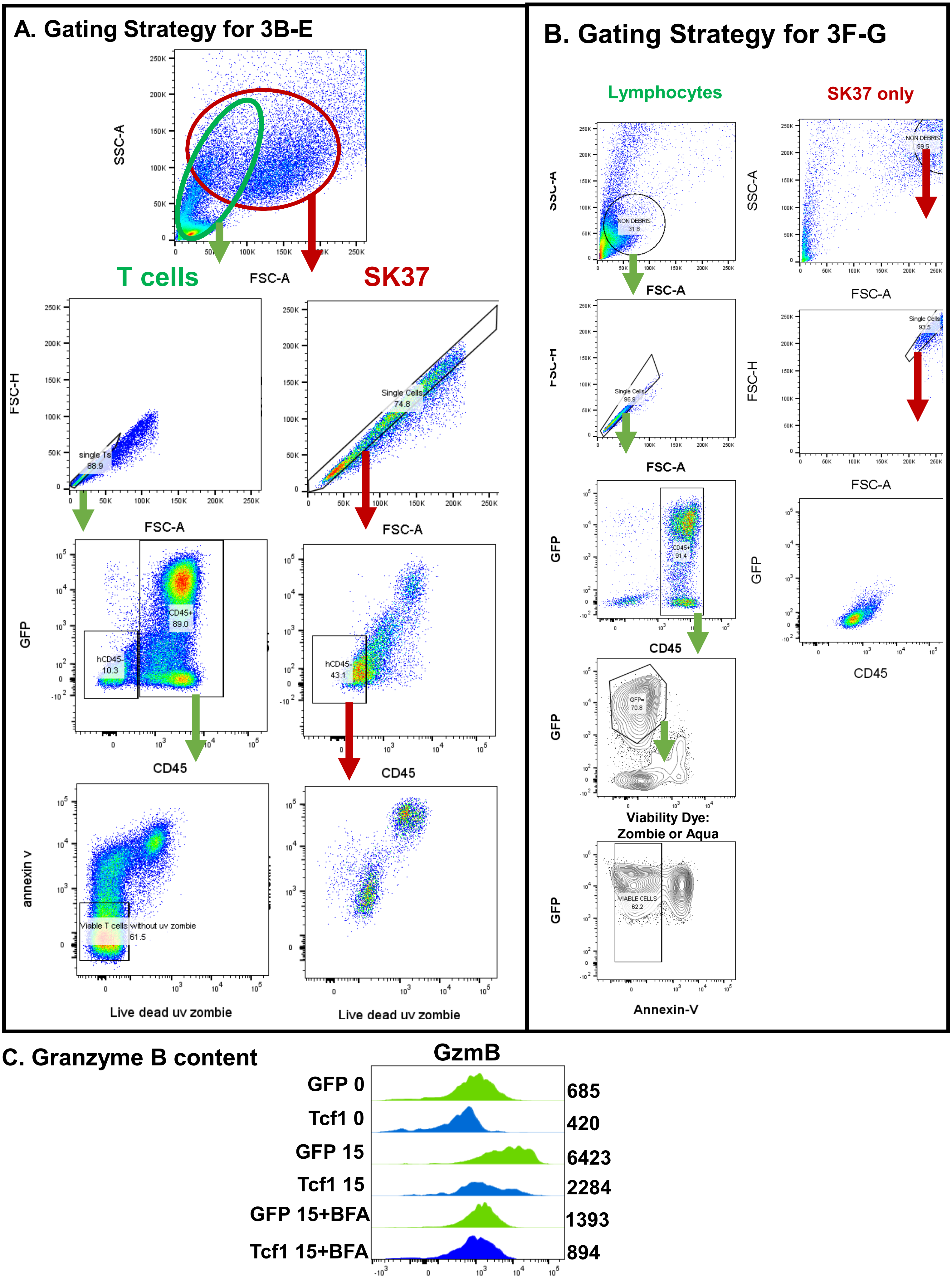

Supplemental Figure 3: Population-Kinetics assays

A. Gating Strategy to assay cell numbers

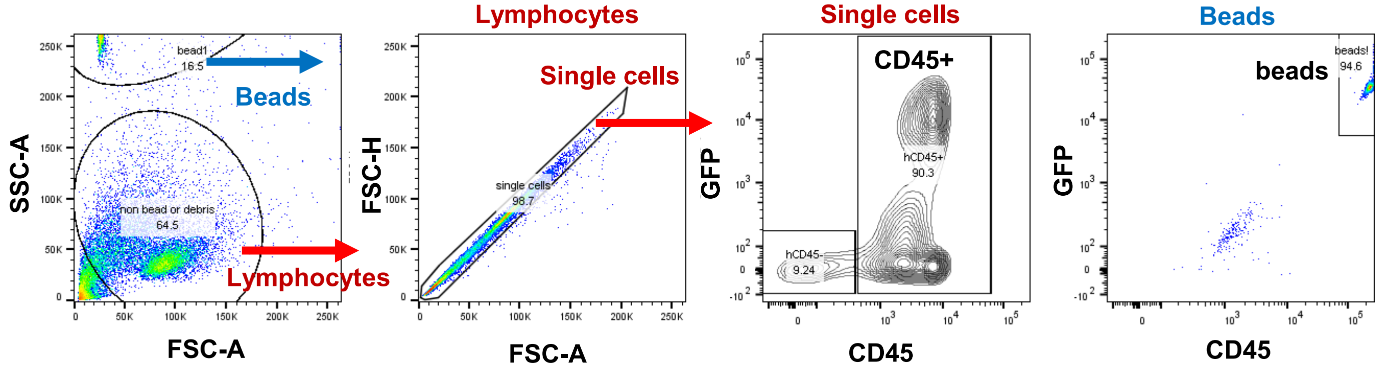

B. Gating Strategy to assay MitoTrackerDeepRed FM labelling at 10nM

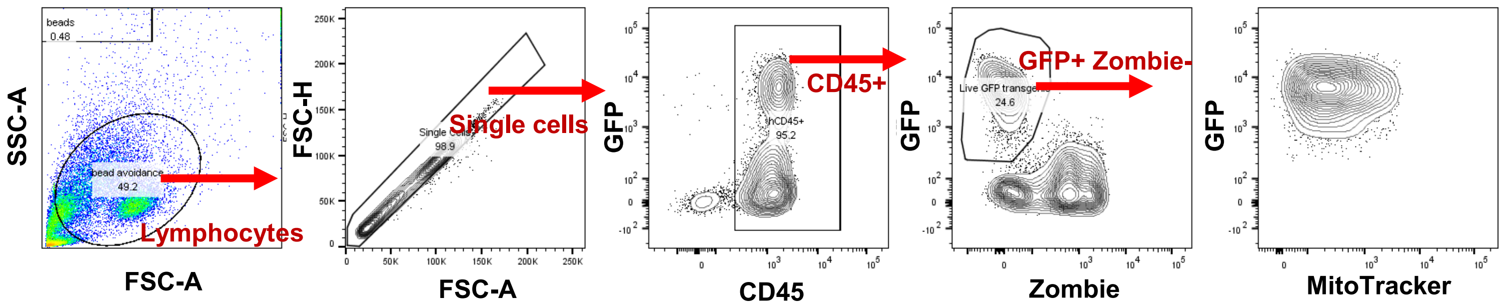

C. Gating Strategy to determine Ki67 labelling D

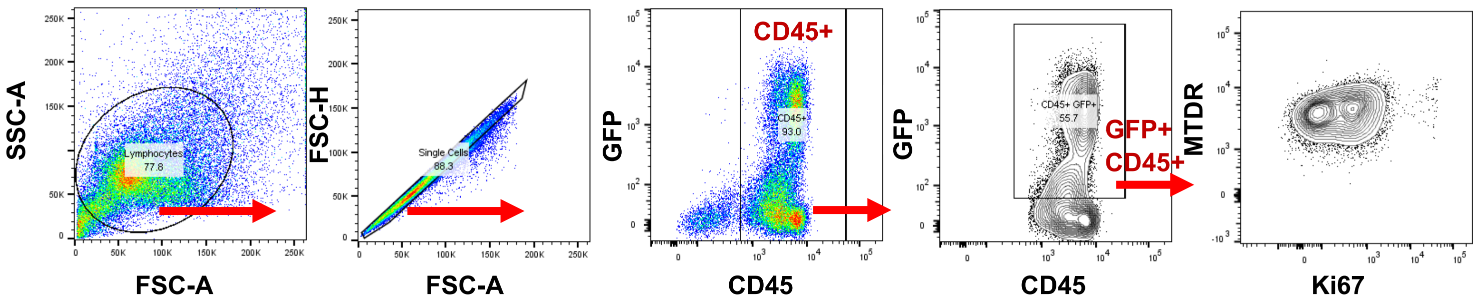

Supplemental Figure 4: Characterization of cells used in model of adoptive transfer

A. CD8 purity and 19305 expression of CD8 T cells, also viewed in Figure 3A

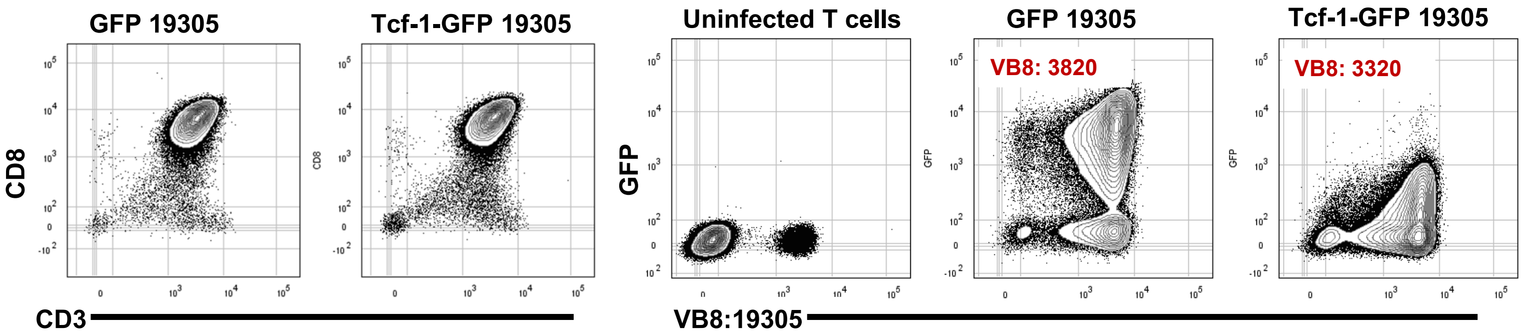

B. Gating Strategy for tumor-infiltrating T cells seen without Live/Dead discrimination

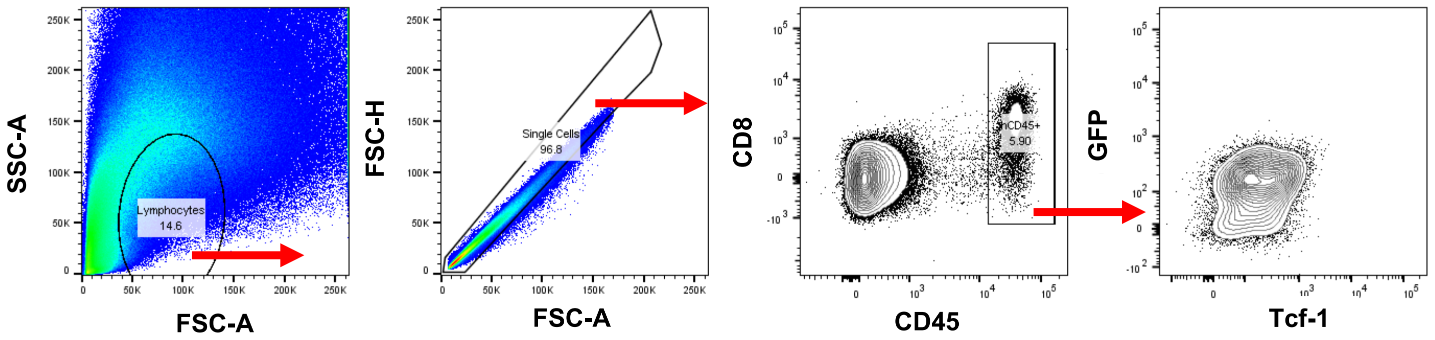

C. Phenotype of Tumor-infiltrating T cells

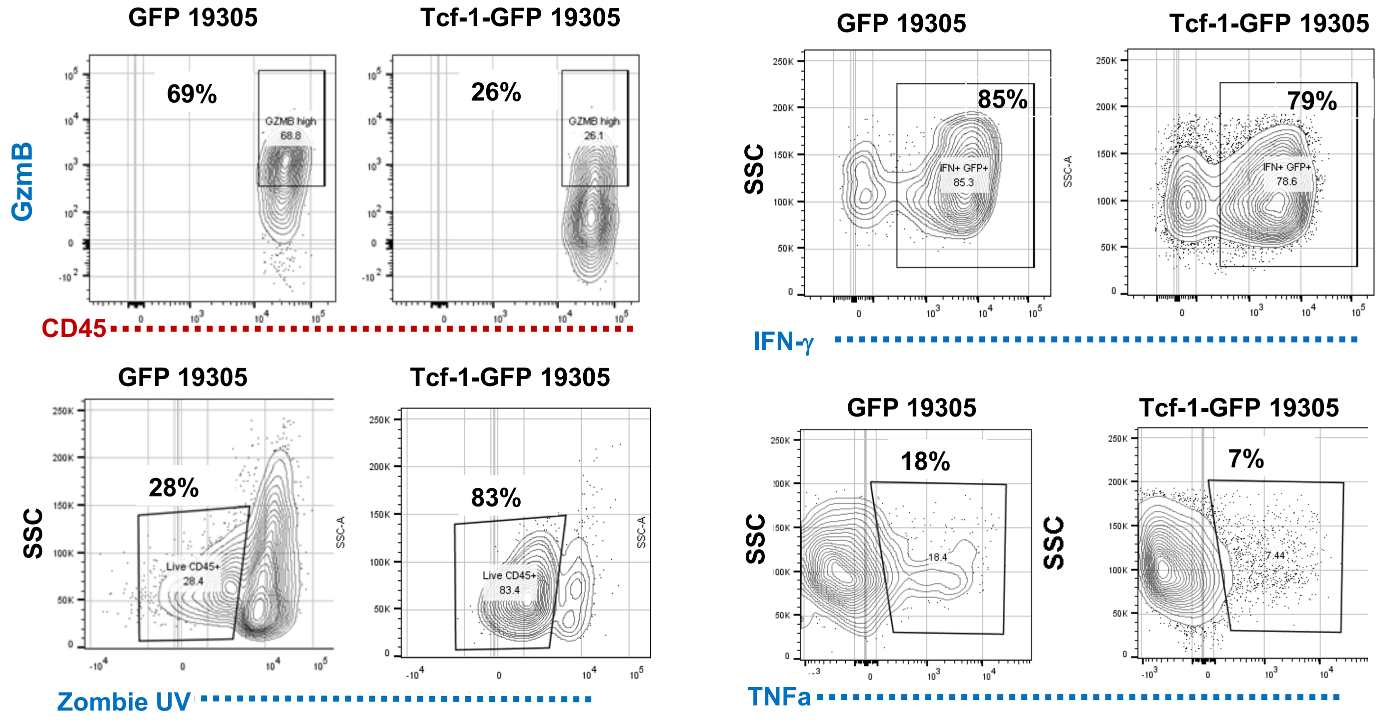

Supplement: Supplementary file 1 — Supplementary file1 (PDF 2630 KB) [file 262_2022_3197_MOESM1_ESM.pdf]
